# Supplementary material for: Classification of images of bee pollen according to their producers
Source: PLoS One. 2025 Oct 21;20(10):e0334615. doi: 10.1371/journal.pone.0334615 (PMC12539701; doi:10.1371/journal.pone.0334615)
Supplement: Attachment — Submitted filename: PONE-D-24-51855_reviewer.pdf [file pone.0334615.s001.pdf]

PLOS ONE

Classification of images of bee pollen according to their producers

--Manuscript Draft--

**Manuscript Number:**	PONE-D-24-51855
**Article Type:**	Research Article
**Full Title:**	Classification of images of bee pollen according to their producers
**Short Title:**	Classification of images of bee pollen according to their producers
**Corresponding Author:**	Francisco Gómez, PhD<br>Universidad Nacional de Colombia - Sede Bogotá: Universidad Nacional de Colombia<br>Bogotá, Bogotá COLOMBIA
**Keywords:**	Corbicular Pollen; Digital Images; Denomination of Origin; Pollen Color; Machine learning
**Abstract:**	The food industry is witnessing a growing interest in pollen due to its nutritional and energy composition. Consumers of bee pollen are increasingly eager to learn about the origins of the products they purchase. Establishing the geographical origin and the producer of pollen can enhance the product's value and meet consumer demands for transparency in the supply chain. This article presents a novel approach for the classification of images of bee pollen according to their producers using digital images and machine learning. The study focuses on pollen collected from various beekeepers in the Boyacá region of Colombia. A standardized image acquisition process was employed to capture macroscopic images of the pollen samples. These images were then analyzed to extract color information, and machine learning models were trained to predict the producer of the pollen based on its color characteristics. The results demonstrate that the proposed approach can effectively determine the producer of pollen samples based on their color information. The model achieved an accuracy rate of 85% in associating pollen samples with their respective beekeepers. This outcome has significant implications for traceability and transparency in the bee pollen industry, offering a cost-effective and accessible method to verify the product's origin.
**Order of Authors:**	Juan Leal-Campuzano, MSc
	Carlos Martínez, PhD
	Francisco Gómez, PhD
**Additional Information:**	
**Question**	**Response**
**Financial Disclosure**<br><br>Enter a financial disclosure statement that describes the sources of funding for the work included in this submission. Review the submission guidelines for detailed requirements. View published research articles from *PLOS ONE* for specific examples.<br><br>This statement is required for submission and **will appear in the published article** if the submission is accepted. Please make sure it is accurate.	The author(s) received no specific funding for this work.

Powered by Editorial Manager® and ProduXion Manager® from Aries Systems Corporation

**Funded studies**<br>Enter a statement with the following details:<br>• Initials of the authors who received each award<br>• Grant numbers awarded to each author<br>• The full name of each funder<br>• URL of each funder website<br>• Did the sponsors or funders play any role in the study design, data collection and analysis, decision to publish, or preparation of the manuscript?<br><br>Did you receive funding for this work?	
**Competing Interests**<br><br>Use the instructions below to enter a competing interest statement for this submission. On behalf of all authors, disclose any competing interests that could be perceived to bias this work—acknowledging all financial support and any other relevant financial or non-financial competing interests.<br><br>This statement is required for submission and **will appear in the published article** if the submission is accepted. Please make sure it is accurate and that any funding sources listed in your Funding Information later in the submission form are also declared in your Financial Disclosure statement.<br><br>View published research articles from *PLOS ONE* for specific examples.	NO authors have competing interests

**NO authors have competing interests**<br><br>Enter: *The authors have declared that no competing interests exist.*<br><br>**Authors with competing interests**<br><br>Enter competing interest details beginning with this statement:<br><br>*I have read the journal's policy and the authors of this manuscript have the following competing interests: [insert competing interests here]*<br><br>* typeset	
**Ethics Statement**<br><br>Enter an ethics statement for this submission. This statement is required if the study involved:<br><br>• Human participants<br>• Human specimens or tissue<br>• Vertebrate animals or cephalopods<br>• Vertebrate embryos or tissues<br>• Field research<br><br>Write "N/A" if the submission does not require an ethics statement.<br><br>General guidance is provided below. Consult the submission guidelines for detailed instructions. **Make sure that all information entered here is included in the Methods section of the manuscript.**	N/A

Powered by Editorial Manager® and ProduXion Manager® from Aries Systems Corporation

**Format for specific study types**<br><br>**Human Subject Research (involving human participants and/or tissue)**<br>• Give the name of the institutional review board or ethics committee that approved the study<br>• Include the approval number and/or a statement indicating approval of this research<br>• Indicate the form of consent obtained (written/oral) or the reason that consent was not obtained (e.g. the data were analyzed anonymously)<br><br>**Animal Research (involving vertebrate animals, embryos or tissues)**<br>• Provide the name of the Institutional Animal Care and Use Committee (IACUC) or other relevant ethics board that reviewed the study protocol, and indicate whether they approved this research or granted a formal waiver of ethical approval<br>• Include an approval number if one was obtained<br>• If the study involved *non-human primates*, add *additional details* about animal welfare and steps taken to ameliorate suffering<br>• If anesthesia, euthanasia, or any kind of animal sacrifice is part of the study, include briefly which substances and/or methods were applied<br><br>**Field Research**<br><br>Include the following details if this study involves the collection of plant, animal, or other materials from a natural setting:<br>• Field permit number<br>• Name of the institution or relevant body that granted permission	
**Data Availability**<br><br>Authors are required to make all data underlying the findings described fully available, without restriction, and from the time of publication. PLOS allows rare exceptions to address legal and ethical concerns. See the PLOS Data Policy and FAQ for detailed information.	Yes - all data are fully available without restriction

A Data Availability Statement describing where the data can be found is required at submission. Your answers to this question constitute the Data Availability Statement and **will be published in the article**, if accepted.<br><br>**Important:** Stating 'data available on request from the author' is not sufficient. If your data are only available upon request, select 'No' for the first question and explain your exceptional situation in the text box.<br><br>Do the authors confirm that all data underlying the findings described in their manuscript are fully available without restriction?	
**Describe where the data may be found in full sentences. If you are copying our sample text, replace any instances of XXX with the appropriate details.**<br><br>• If the data are **held or will be held in a public repository**, include URLs, accession numbers or DOIs. If this information will only be available after acceptance, indicate this by ticking the box below. For example: *All XXX files are available from the XXX database (accession number(s) XXX, XXX.*).<br>• If the data are all contained **within the manuscript and/or Supporting Information files**, enter the following: *All relevant data are within the manuscript and its Supporting Information files.*<br>• If neither of these applies but you are able to provide **details of access elsewhere**, with or without limitations, please do so. For example:<br><br>*Data cannot be shared publicly because of [XXX]. Data are available from the XXX Institutional Data Access / Ethics Committee (contact via XXX) for researchers who meet the criteria for access to confidential data.*<br><br>*The data underlying the results presented in the study are available from (include the name of the third party*	The data used in this study are available from the authors upon reasonable request

and contact information or URL).<br>• This text is appropriate if the data are owned by a third party and authors do not have permission to share the data.<br><br>* typeset	
Additional data availability information:	Tick here if the URLs/accession numbers/DOIs will be available only after acceptance of the manuscript for publication so that we can ensure their inclusion before publication.

Powered by Editorial Manager® and ProduXion Manager® from Aries Systems Corporation

Manuscript

Click here to access/download;Manuscript;PollenFinalVersion.pdf

Classification of images of bee pollen according to their producers

Juan Leal-Campuzano[1,2], Carlos A. Martínez[3], Francisco Gómez[4*]

[1]Dirección de Investigación y Desarrollo, Corporación Colombiana de Investigación Agropecuaria - AGROSAVIA, Madrid, Cundinamarca,Colombia.
[2]Facultad de Sistemas, Escuela Tecnológica Instituto Técnico Central, Bogotá,Colombia.
[3]Departamento de Producción Animal, Facultad de Medicina Veterinaria y de Zootecnia, Universidad Nacional de Colombia, Bogotá, Colombia.
[4*]Departamento de Matemáticas, Facultad de Ciencias, Universidad Nacional de Colombia, Bogotá, Colombia.

*Corresponding author(s). E-mail(s): fagomezj@unal.edu.co;

Abstract

The food industry is witnessing a growing interest in pollen due to its nutritional and energy composition. Consumers of bee pollen are increasingly eager to learn about the origins of the products they purchase. Establishing the geographical origin and the producer of pollen can enhance the product's value and meet consumer demands for transparency in the supply chain. This article presents a novel approach for the classification of images of bee pollen according to their producers using digital images and machine learning. The study focuses on pollen collected from various beekeepers in the Boyacá region of Colombia. A standardized image acquisition process was employed to capture macroscopic images of the pollen samples. These images were then analyzed to extract color information, and machine learning models were trained to predict the producer of the pollen based on its color characteristics. The results demonstrate that the proposed approach can effectively determine the producer of pollen samples based on their color information. The model achieved an accuracy rate of **85%** in associating pollen samples with their respective beekeepers. This outcome has significant implications for traceability and transparency in the bee pollen industry, offering a cost-effective and accessible method to verify the product's origin.

Keywords: Corbicular Pollen, Digital Images, Denomination of Origin, Pollen Color, Machine Learning

1 Introduction

The food industry is increasingly interested in pollen due to its nutritional and energy composition (Kostić et al, 2020). Bee pollen has also piqued the interest of the pharmaceutical and personal care industries (Kurek-Górecka et al, 2020). On the other hand, consumers are increasingly eager to learn more about the origin and production processes of the products they purchase (Kieliszek et al, 2018). For example, the *seal of origin*, which identifies unique products from specific regions, demonstrates how geographical product origin can be relevant to consumers (Katerinopoulou et al, 2020). In recent years, various beekeeping-based products have earned these seals of origin (Galhardo et al, 2020; Silva et al, 2021). However, the consumer demand for transparent supply chain practices continues to challenge the bee pollen industry to provide visibility and disclose this information to consumers (Kraft et al, 2018).

The development of beekeeping has positive agricultural impacts (Klein et al, 2007). For instance, approximately 70% of crops experience increased production when pollinators visit their flowers (Wilcock and Neiland, 2002). Worldwide, about 12 pollinator species are commercialized, with *Apis mellifera* and *Bombus terrestris* being the most widespread (Porto et al, 2020). Most apiaries primarily focus on honey production using these pollinators; however, in regions where environmental conditions support diverse pollen flora, such as in some developing countries (Thakur and Nanda, 2020), pollen collection and commercialization have become significant economic activities (Thakur and Nanda, 2020). Nonetheless, there is a need for improved mechanisms to add value to the pollen production chain, particularly in developing countries (Thakur and Nanda, 2020).

One way to meet customer requirements and enhance pollen production and commercialization is by linking the product to its geographical origin (Luykx and Van Ruth, 2008). Associating a product with its origin connects information about the product, including its reputation, quality, and nutritional composition, with the production location and the producer, ensuring high-quality products and increasing their economic value (Katerinopoulou et al, 2020). However, implementing this added value mechanism requires addressing the challenge of determining the geographical origin of pollen, i.e., determining the origin of a given pollen sample (Dimou et al, 2007; Uršulin-Trstenjak et al, 2017).

Bees exhibit floral fidelity, meaning they prefer specific floral species (Sanderson and Wells, 2005), particularly those that provide the most nutrients for the hive (Waser, 1986). Consequently, pollen may encode geographic information through its botanical origin. Based on this observation, various methods have been proposed to address the problem of determining the geographical origin of pollen. Palynology and bromatology are the most widely used methods for establishing pollen's geographical origin (Agashe, 2006). However, these methods are time-consuming and require specialized training, which is not readily available in productive areas of developing

countries (Nates-Parra et al, 2013). Physicochemical processes are also used for pollen origin characterization (Prdun et al, 2021), but these methods require expensive and specialized equipment that is inaccessible to many participants in the pollen production chain (Kostić et al, 2020). More recently, computer vision has emerged as an alternative for assessing certain pollen properties, such as its composition (Rodriguez-Damian et al, 2006; Lunau, 1995; Dhawale and Tidke, 2013). Nevertheless, most of these approaches focus on microscopic images, which are challenging to acquire and analyze, limiting their use in determining the geographical origin of pollen. Thus, establishing the geographical origin of pollen samples using cost-effective methods presents a significant challenge in building a value-added product pipeline.

This work proposes a novel strategy for the classification of images of bee pollen according to their producers using digital images acquired under semi-controlled conditions and machine learning-based methods. It is worth remarking that once the producer is identified, its location can be linked to the geographical location, providing an alternative way to characterize the spatial pollen origin. The method links the color composition of macroscopic pollen images, i.e., images taken at a real-life scale, to the producer of the sample using machine learning. This approach is more accessible and cost-effective as compared to previous methods that relied on specialized acquisition devices and highly trained experts. To achieve this, a color segmentation approach for pollen and a supervised machine-learning strategy for automatically determining the origin of apiary pollen was used. The primary contribution of this work is providing evidence, for the first time, that macroscopic pollen images can also offer information about color composition related to the producer and its geographical origin. This discovery opens up new possibilities for studying other pollen properties under simpler acquisition conditions, including pollen nutritional composition and other tasks required by the pollen production chain.

2 Materials and methods

Figure 1 illustrates the proposed approach for the classification of macroscopic images of bee pollen according to their producers. The process begins with the collection and preparation of samples from a group of pollen producers. Subsequently, a standardized acquisition process generates a set of pollen sample images. Afterwards, a pre-processing stage is employed to enhance the image quality. Color characteristics then serve as a discriminative representation of the pollen composition. A machine learning classification model, based on the producer's location, is used to link the image's color information to the apiary where the pollen is produced. This model is evaluated by predicting the pollen producer from images acquired under the same conditions.

2.1 Pollen samples

This study involved beekeepers from various locations of the Márquez region, including the municipalities of Nuevo Colón, Turmequé, Viracachá, and Ciénega, as well as the Tundama region, encompassing the municipalities of Tibasosa, Tuta, Belén, Santa Rosa de Viterbo, Duitama, Paipa, and Tuta, all located within the Boyacá department

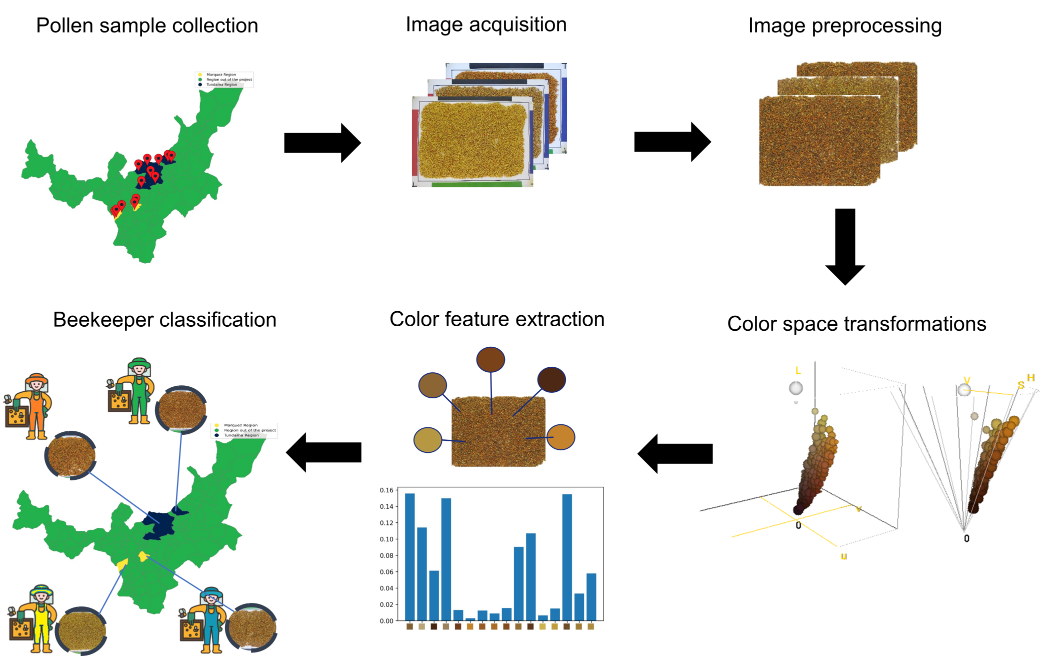

Fig. 1 *Identification of the geographic origin of the pollen begins with collecting a set of pollen samples from various regions. Macroscopic pollen images are acquired under standardized conditions. A pre-processing process is applied to enhance the images, followed by extracting color information from the images. A machine learning-based classification method is then used to establish the connection between color information and the pollen producer.*

of Colombia. These municipalities are located in regions known for high pollen production. Samples were collected in 2021 from paramo and humid ecosystems during the flowering season to capture potential variations in bee preferences. A total of twenty producers participated in the study, with each producer providing five pollen samples collected from three different hives. This process was repeated four times throughout the year, with variations in the hives selected, resulting in a total of 60 pollen samples per producer. Each pollen sample weighed 200 grams, with 100 grams designated for image acquisition. The pollen samples were dried to achieve a humidity level of 4% and maintained at a temperature of 55°C to facilitate grain manipulation. It's important to note that environmental conditions can influence bee behavior (Devkota et al, 2022). This study did not require ethics approval as it did not involve human participants, animal subjects, or any data that would necessitate ethical oversight.

2.2 Sample image acquisition

Images were acquired under standardized conditions to reduce variability in illumination and preserve color information. Two separate image acquisitions, each consisting of two images, were performed at two different times by two researchers to assess the

reliability of the approach. The acquisition protocol involved using a lightbox to capture images of pollen samples under controlled lighting and geometric conditions at a macroscopic scale. The acquisition device was equipped with an LED-based lighting system and a matching interior cover designed to minimize reflections and reflectance artifacts in the acquired images. The dimensions of the device and the camera position were adjusted to optimize the field of view for the pollen sample located at the bottom. Additionally, the lightbox featured a reference color mark with red, green, blue, and black guidelines placed on the box's base, which was later used for standardizing the colors in the images. Figure 2 displays the device utilized for capturing images of the pollen samples.

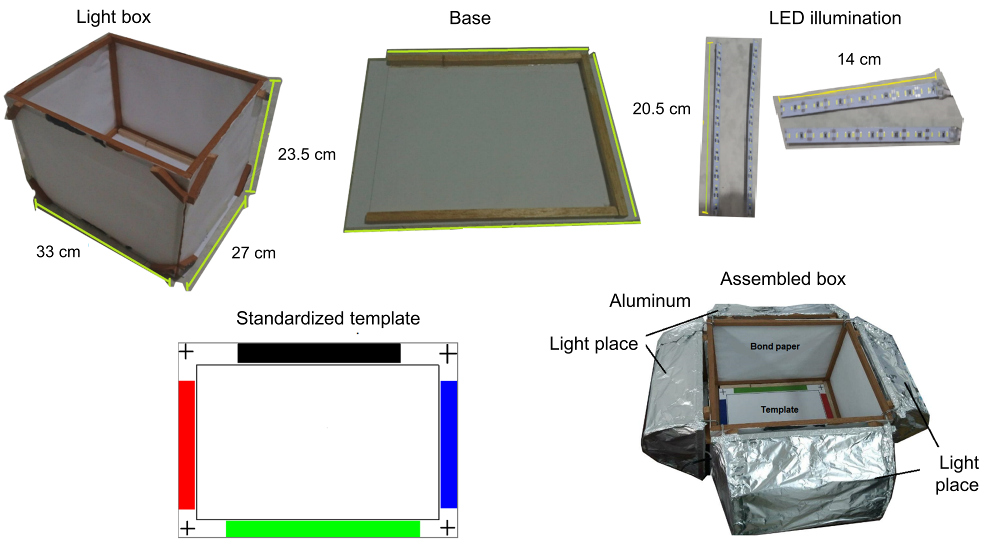

Fig. 2 *Lightbox utilized to standardize the acquisition of pollen sample images at a macroscopic scale. The device was equipped with an LED-based illumination system and a matte interior, which aided in maintaining standardized lighting and geometric conditions.*

The grains were evenly spread across the template at the bottom of the lightbox to capture images of the pollen samples. These images were captured using a Xiaomi Redmi 9 mobile camera with a resolution of 1800 × 2400 pixels. Four images were acquired for each pollen sample. Figure 3 illustrates an image corresponding to a pollen sample captured using the proposed method.

2.3 Image preprocessing

An algorithm based on local entropy texture was used to segment the image area containing pollen grains (Wang et al, 2020). Specifically, the entropy of the local

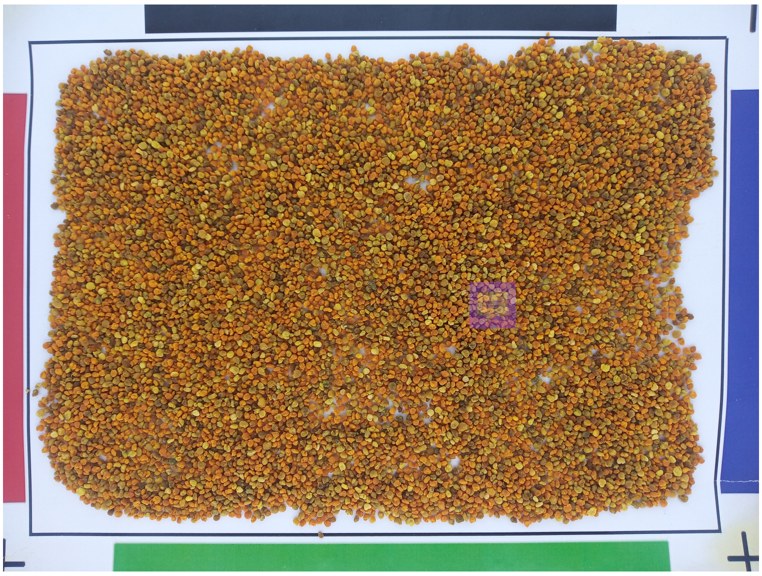

Fig. 3 *Image pollen sample acquired under the proposed acquisition protocol.*

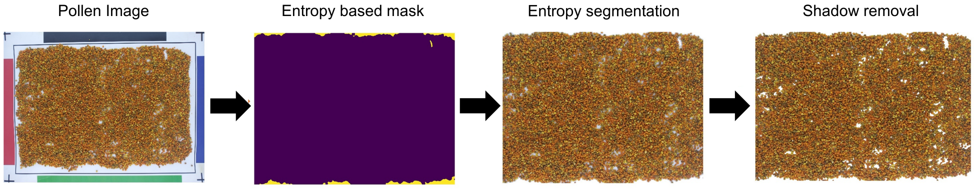

Fig. 4 *Pre-processing of pollen images, from digitization on the left to shadow removal on the right.*

histogram of gray levels in circular patches with a radius of five pixels was calculated. Subsequently, the Otsu binarization algorithm was applied to determine a threshold for separating the pollen (high entropy) from the background (low entropy) (Otsu, 1979).

Additional post-processing was performed to eliminate any extra noisy pixels associated with small grain shadows in the image. To achieve this, the distance between the RGB color of each pixel and the diagonal of the RGB cube, which distinguishes different gray levels in an image within the RGB cube, was computed as:

$$\frac{\|U \times P\|}{\|U\|} \tag{1}$$

where $U = [1, 1, 1]$ represents the direction vector associated with the cube's diagonal line, and P represents the RGB color triple. Once the distances for all the pixels were calculated, pixels with distances less than one standard deviation from the cube's diagonal line were considered part of the region of interest. Figure 4 illustrates the cleaning process applied to a pollen image.

2.4 Color space transformation

Color serves as a natural perceptual marker for characterizing pollen. The chemical compounds responsible for pollen color include pigments such as flavonoids and carotenoids (Lunau, 1995). These compounds can vary in quantity and chemical interactions within each pollen species, resulting in distinct color variations. Consequently, a relationship exists between the color of the pollen and the species from which it originates (Gray and Boucot, 1975). This study leverages the color information of pollen grain images to create a distinctive feature associated with the geographical location.

To capture the color characteristics associated with the pollen producer, the color initially represented in the RGB color space was transformed into more suitable color representations. A color space is a multidimensional representation of the color spectrum (Dubois, 2010). For this study, two color spaces were considered for characterization: HSV (Hue, Saturation, and Value) and $L^*u^*v^*$ of the CIE (Commission Internationale de l' Éclairage) (Dubois, 2010).

The HSV color space is designed to separate the luma (value) from the color information (hue and saturation). This representation is invariant to variations in light intensities and shadows, which can otherwise distort the color information, and allows for a clear description of the color characteristics in the pollen grains (Vadivel et al, 2005). The conversion of colors for each pixel from the RGB space to the HSV space was carried out as follows:

$$H = \arctan\left(\frac{\sqrt{3}(G-B)}{2R-G-B}\right) \tag{2}$$

$$S = \begin{cases} 0 & if \quad C = 0 \\ \frac{C}{V} & otherwise. \end{cases} \tag{3}$$

$$V = \max(R, G, B) \tag{4}$$

where $C = \max(R, G, B) - \min(R, G, B)$.

The $L^*u^*v^*$ color space offers a perceptually uniform color characterization, meaning that color differences computed in this space are proportional to how humans perceive these differences (Paschos, 2001; Burger and Burger, 2009; Vadivel et al, 2005). In the $L^*u^*v^*$ color space, colors that appear similar in pollen are situated closely regarding their distances. At the same time, pixels perceived as different by human perception are positioned farther apart. This representation enhances the intraclass/interclass variability for similar/different colors. The transformation of pixels from RGB to the $L^*u^*v^*$ color space was carried out as follows:

$$\begin{pmatrix} X \\ Y \\ Z \end{pmatrix} = \begin{pmatrix} 0.607 & 0.174 & 0.2 \\ 0.299 & 0.587 & 0.114 \\ 0 & 0.066 & 1.116 \end{pmatrix} \begin{pmatrix} R \\ G \\ B \end{pmatrix} \tag{5}$$

followed by a nonlinear transformation that takes into consideration the standard values for the white color (Y_0, u_0, v_0) according to the CIE, as follows:

$$L = \begin{cases} 116\sqrt[3]{Y} - 16 & for \quad Y > 0.008856 \\ 903.3 \cdot Y & for \quad Y \leq 0.008856 \end{cases} \tag{6}$$

$$u^* = 13L\left(\frac{4X}{X + 15Y + 3Z} - u_0\right) where \quad u_0 = 0.19793943 \tag{7}$$

$$v^* = 13L\left(\frac{6Y}{X + 15Y + 3Z} - v_0\right) where \quad v_0 = 0.46831096. \tag{8}$$

The original RGB images were characterized using four representations: HSV, L*u*v, HS, and *u*v, to investigate the hypothesis that color is a highly informative feature for this problem. It is worth noting that the last two representations only considered color information.

2.5 Color feature extraction

Once suitable representation spaces had captured the pollen color information, a set of low-dimensional features was constructed to be used as input for machine learning algorithms. To achieve this, a color quantization algorithm was applied to generate a compact set of representative colors for the pollen grains under study (Celebi, 2011). The k-means clustering algorithm was used to identify the most representative colors in the pollen images. To address the computational challenge posed by the large number of pixels in all the pollen images, a k-means mini-batch algorithm was employed (Arthur and Vassilvitskii, 2006; Sculley, 2010). This algorithm divides the clustering process into smaller batches, improving processing times. The number of species identified in an independent palynological analysis conducted on the collected pollen samples determined the number of centroids for the k-means algorithm. The palynological study reported between 16 to 35 different species per sample. Therefore, the k parameter also ranged from 16 to 35. Each pixel was assigned to the nearest representative color determined by the k-means algorithm based on the minimum Euclidean distance. Subsequently, each image was represented by a k-dimensional vector containing the frequencies of occurrence of the k key colors within the image. This representation was calculated for all four color spaces under investigation. Figure 5 illustrates the different feature vectors obtained for the various color spaces computed for a sample.

2.6 Classification of the pollen producer

Utilizing feature vectors that describe the color information of the pollen, a multiclass classification strategy based on machine learning was employed to determine the pollen's producer and, subsequently, its geographical location. Specifically, a set of classifiers was created, with color feature vectors as input and the identification of the beekeeper as the output. Various multiclass classification strategies were explored, including support vector machines with polynomial kernels with degrees varying between 6 and 9, random forest with 200 trees in the forest, and a multilayer perceptron with 64 neurons in the hidden layer (Russell, 2010).

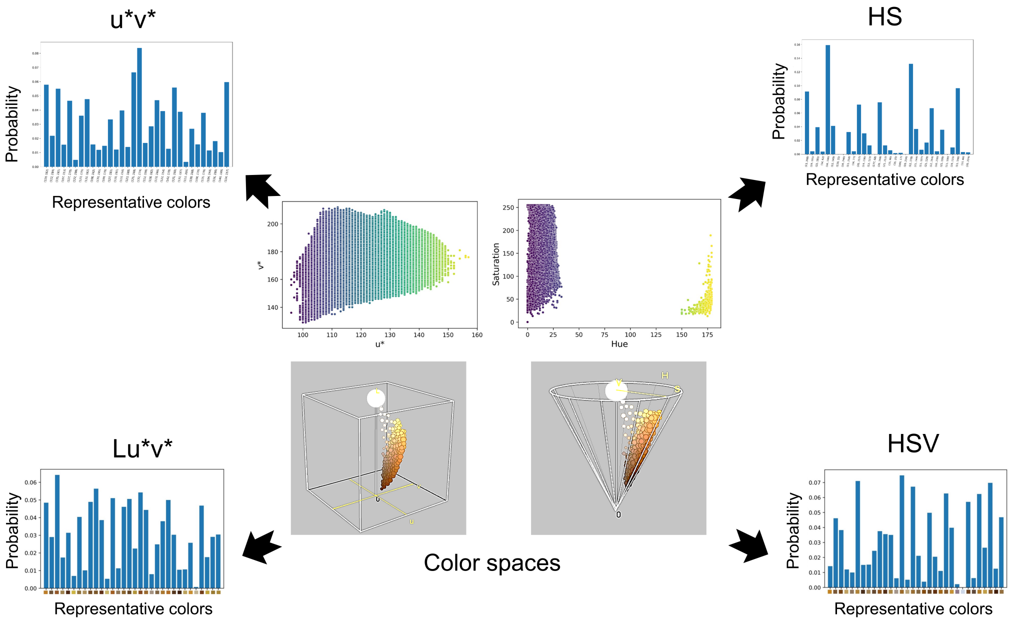

Fig. 5 *Different histograms represent characteristic vectors for each color space.*

2.7 Experimental evaluation

The experimental setting aimed to quantify the capacity of the proposed strategy to correctly determining the beekeepers using the images of pollen samples acquired under the proposed protocol.

According to the methodological proposal, a total of 4800 images were expected. However, due to practical difficulties related to adverse weather conditions in the field, the number of collected samples was reduced to 4572. This reduction resulted in a slight class imbalance, later accounted for in the classifiers' training and validation stages. A total of 3048 images were considered in the training and validation sets, and 1524 pollen images in the test set to evaluate the classifier's performance. Each machine learning strategy was trained using the four different color representations, i.e., each color representation resulted in a different classifier. For each classifier, different hyperparameters (or tuning parameters) were also considered. A stratified cross-validation procedure selected the best classification model and the corresponding hyperparameters (Zhang et al, 2016). This data partition strategy is a variation of the k-fold that returns stratified folds. In this case, ten folds were conformed to preserve the percentage of samples for each class. This strategy ensures the training and validation sets have the same proportions of beekeepers as in the original dataset.

The predictive performance was evaluated using the multiclass confusion matrix with accuracy, precision, recall, and F1-score as parameters informing about predictive capability. The macro-precision, macro-recall, and macro F1-score were also computed for the evaluation. The weighted-average F1 score, calculated as the weighed mean of

all per-class F1-scores using the number of samples per class as weights, was used for model selection in the validation partition (Grandini et al, 2020).

The repeatability, also called test–retest reliability, of the proposed method for the beekeeper association task was also studied (Waugh and Clark, 2021). In this case, the test-retest reliability refers to the consistency of the proposed classification system when the same pollen sample is classified multiple times under the same conditions but at different points in time. This performance measurement assesses how well the classification results, i.e., the pollen producer assignment, can be reproduced if the test is repeated on a different pollen image sample. The goal is to ensure the beekeeper classification method is stable over time and yields similar results when the same data is classified again. For this evaluation, the test partition for each beekeeper was divided into two subsets taken at different times and containing the same number of images per beekeeper. Then, the trained classifier was used on the first data set obtained at one point to compute individual F1 scores for each beekeeper. Then, the second set of data corresponding to a different point in time was used also to compute these F1 scores. Finally, the repeatability between the first and the second dataset ($r_{1,2}$) was calculated as the Pearson correlation coefficient between the individual F1 scores obtained for each beekeeper in the two test partitions, as follows:

$$r_{1,2} = \frac{\sum_{i=1}^{n}(f_{i,1} - \bar{f}_1)(f_{i,2} - \bar{f}_2)}{\sqrt{\sum_{i=1}^{n}(f_{i,1} - \bar{f}_1)^2}\sqrt{\sum_{i=1}^{n}(f_{i,2} - \bar{f}_2)^2}}$$

where $f_{i,1}$ is the F1 individual score for obtained for the i-th pollen producer on the first dataset, $f_{i,2}$ the F1 score for obtained for the i-th pollen producer on the second dataset, $\bar{f}_1$ is the mean of the F1 individual scores for all beekeepers in the first dataset, $\bar{f}_2$ is the mean of the F1 individual scores for all beekeepers in the second dataset and n is the number of beekeepers.

3 Results

Figure 6 exemplifies beekeeper associations provided by the proposed model for pollen images. In the figure, the project's relevant regions are highlighted in blue and yellow. As evident, the proposed model correctly associates beekeepers with the majority of the pollen images used as input. However, the red arrow in the figure indicates an erroneous association between two pollen producers located in the municipality of Duitama (Boyacá). In this instance, the proposed model made an incorrect prediction, possibly due to the geographical proximity of both producers.

Figure 7 shows the confusion matrix obtained by applying the proposed strategy to a test partition involving twenty pollen producers. The municipalities where the beekeepers are located are listed in the rows of the first column of the figure. These results correspond to the model with the highest performance in the weighted-average F1-score, specifically a support vector machine trained using a feature vector consisting of 32 reference colors resulting from clustering in the uv color space. The identified model achieved an accuracy of 85% in the beekeeper association task using pollen images.

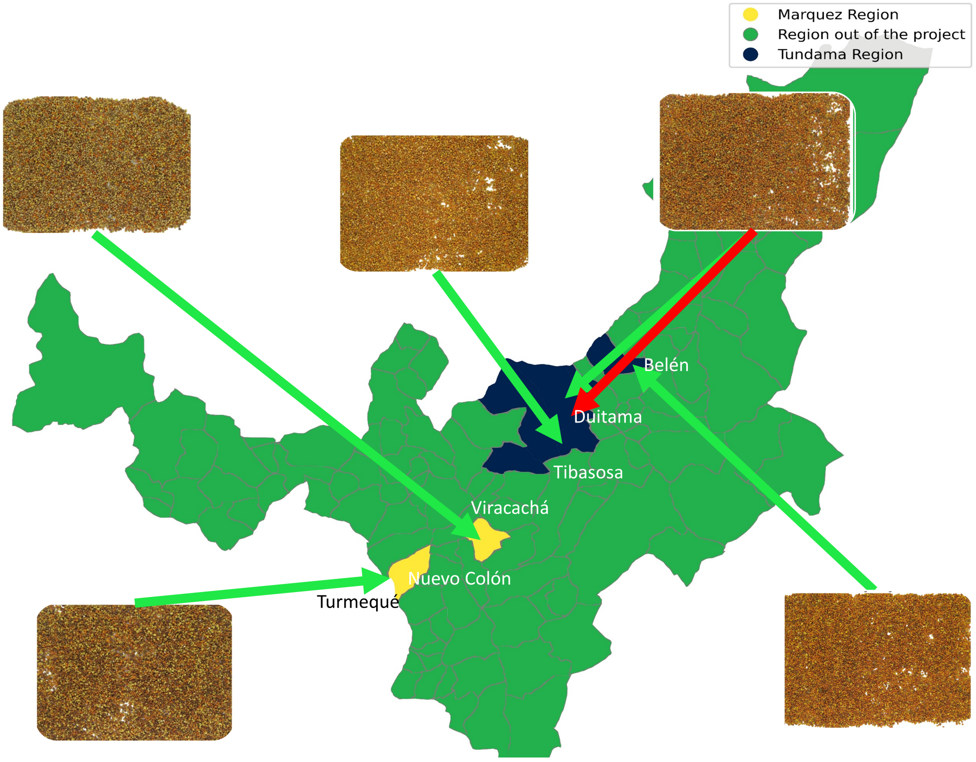

Fig. 6 *Examples of beekeeper associations resulting from using pollen images. Regions involved in the project (blue and yellow) and some pollen images. Green arrows indicate correct beekeeper identifications and the red arrow an incorrect association.*

As observed, the diagonal of the confusion matrix demonstrates that the proposed approach performs well. Notably, the method resulted in a macro-average recall of 84%, with individual recalls exceeding 80% for most beekeepers (15 out of 20), indicating the model's capability to identify instances of specific beekeepers correctly. Similarly, the macro-average precision was 85%, with individual precisions exceeding 80% for 17 out of 20 beekeepers, highlighting the model's capacity to identify only the pollen samples associated with particular beekeepers correctly. Finally, the total macro F1-score was also 85%, indicating a good balance between precision and recall when considering all beekeepers.

When the beekeeper association model was employed as a proxy to identify the municipality, i.e., the municipality was determined using the identified beekeeper, the method achieved an accuracy of 88%, a macro-average precision of 86%, a macro-average recall of 86%, and an F1-score of 86%. These results indicate strong performance in the task of identifying the location of the producer.

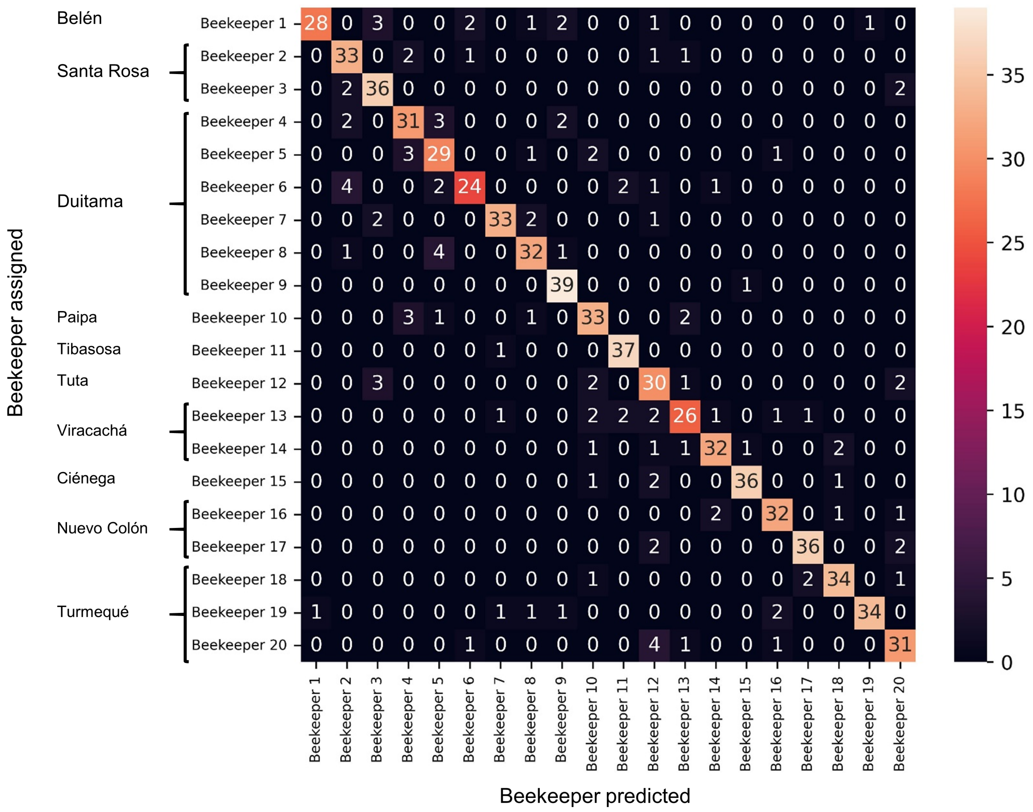

Fig. 7 *Confusion matrix for the pollen producer classification on 762 testing pollen images.*

Figure 8 displays a scatter plot comparing the F1-score performances for the two test partitions involving the twenty producers considered for the test-retest reliability assessment. The figure illustrates the prediction interval, which represents the range of values likely to contain the value of a new observation under specified predictor settings. As observed, the method exhibited highly similar performances in both test partitions, with narrow confidence intervals. In this case, the Pearson correlation ($r_{1,2}$) was 0.83, indicating good reliability. When assessing the test-retest reliability for municipality location, the Pearson correlation was 0.92, which amounts to an excellent reliability (Akoglu, 2018; Koo and Li, 2016).

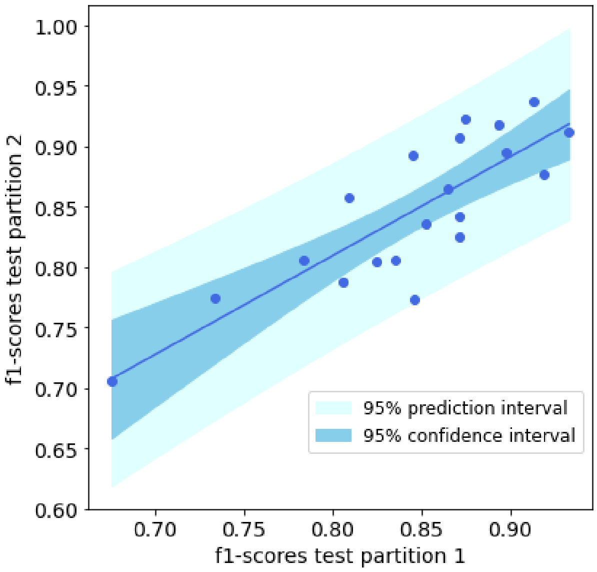

Fig. 8 *Scatter plot between the performances obtained in each beekeeper for two different predictions used to compute the test-restest reliability.*

4 Discussion

This paper introduces a novel machine learning-based strategy for identifying the geographical origin of corbicular pollen samples using macroscopical product images. The approach relies on a standardized protocol for image sample acquisition, suitable for use by various stakeholders in the pollen production chain. These samples allow the construction of a predictive model that links image information and beekeeper identification by leveraging the similarities in the color distributions observed in samples from the same geographical location. In contrast to the traditional approach for characterizing pollen's geographical origin commonly used in palynology, which requires a high level of expertise and expensive equipment, this study provides evidence that easily obtained pollen color image information at a macro scale can still provide valuable insights into pollen origin.

Due to increased globalization in the food trade, ensuring the integrity of the food chain involves challenges such as origin fraud and quality assurance (Aung and Chang, 2014). Consumers increasingly demand tangible traceability proof to assess food quality and safety (Bertolini et al, 2006). There is also a need for traceability systems that provide comprehensive information about food product origin, processing, distribution, and ultimate destination. Associating a product to its origin is fundamental for transparent supply chain management, particularly in developing traceability systems (Opara, 2003). In recent years, the beekeeping industry has experienced a rapid growth, leading to an increased demand for traceability systems in the supply chains (Rünzel et al, 2021; Donnelly et al, 2008; Haefeker, 2021). Currently, most traceability-related evidence associated to a product's origin is provided

by specialized labs. In the case of the emerging pollen industry, access to these facilities still needs to be improved, especially in developing countries. Our results show that machine learning strategies may help to identify the product's origin and even the producer. In particular, identifying the producer resulted in performances of 85% for the F1-score without relying on palynological or bromatological studies, which are often cost-prohibitive for different supply chain actors. Our results also suggest a high discriminative capacity to distinguish among individual locations and pollen producers, as evidenced by the high individual recall and precision values observed for more than 80% of the studied producers (see Figure 7). Notably, the proposed model also achieved high reliability (see Figure 8), indicating that the proposed association strategy can be used repeatedly under the same conditions, yielding consistent results, an essential requirement for designing characterization systems in food systems. Recent works suggest the use of smart food traceability systems that have the potential to significantly improve food safety in global food supply chains (Yu et al, 2022). Our results indicate that this work may contribute to developing these intelligent supply chains.

The proposed model for the classification of images of bee pollen according to their producers relies on color information acquired at a macroscopical level of the product. Pollen color is crucial for product characterization and represents a valuable source for palynological studies (Halbritter et al, 2018; Almeida-Muradian et al, 2014). Our results indicate that pollen colors are not randomly distributed, aligning with previous evidence linked to specific distributions of plant communities and pollinator taxa preferences (Lau and Galloway, 2004; Jorgensen et al, 2006). Interestingly, some of the observed inaccuracies in beekeeper classification were linked to close geographical locations, suggesting a strong relationship between pollen color acquired at the macro level and the geographical location. Recent works on characterizing pollen species using artificial intelligence also rely on color information, showing the potential for automating highly specialized analysis tasks (Mahmood et al, 2023; Tsiknakis et al, 2022; Astolfi et al, 2020). However, these approaches rely on data acquired with a light microscope, which may be unsuitable for inclusion in food traceability systems. Previous works have proposed similar histogram color-based representations for approaching the task of colorimetric description of pollen (Salazar-González et al, 2018). In particular, Salazar-González et al. focus on quantifying the percentage of a set of manually predefined colors present in the sample, evidencing the utility of pixel information for describing the pollen color composition. The proposed approach extends this work by evidencing the possibility of linking this color information to the pollen producer and determining the set of colors describing the pollen automatically. Other machine learning-based approaches have also been explored to predict pollen origin (Gonçalves et al, 2018). However, this approach relies on the composition of physicochemical pollen, which may require highly specialized equipment. In contrast, the proposed method focuses for the first time on a different task, determining the pollen producer, and is based on macroscopical images, which are a less specialized data source for acquisition.

The main objective of this work was to propose an approach to classify the pollen producer out of the color information that macroscopical images of the pollen. For

this reason, the proposed approach critically relied on the pollen's color information, which has been highly informative for similar settings (Halbritter et al, 2018; Almeida-Muradian et al, 2014). Therefore, the main focus of the proposed experimental setting was constructing a robust color-based representation of the pollen information, i.e., creating a highly informative/discriminative representation space for the color information. Importantly, one of the main restrictions for this problem was the limited amount of available data. For this, different considerations were accounted for when building this color representation space on this problem. First, we proposed a controlled acquisition strategy aimed at reducing illumination variability, which likely improved the input data quality. Following a preprocessing strategy was also considered to remove spurious color information, which may also negatively affect representation quality. Finally, the color information was captured by combining a highly informative color representation space and a probability-based presence of color representation based on clustering, helping to represent color information compactly. Our results indicate that for this problem, these particular methodological considerations may help construct a highly informative and discriminative representation space, which can be suitably exploited even for classical machine learning strategies, such as those explored here. Alternatively, more recent approaches, such as those based on deep learning, can also be studied to address this problem (Prince, 2023). Most of these methods rely on automatically constructing the representation space by optimizing the model to adjust large amounts of data. Approaches based on transfer learning can also be explored, but these alternatives need more interpretability and may increase overfitting risk compared to less complex machine learning classification approaches (Prince, 2023). Therefore, further research could focus on expanding the dataset, exploring more advanced deep learning techniques, and investigating the potential of transfer learning in this context.

The present approach has other limitations and potential for improvement. First, the model was tested with a small number of beekeepers in a specific geographical location of the department of Boyacá (Colombia). Future work may involve generalizing the association task to other areas producing pollen. The quality of the results can be highly dependent on the image sample acquisition conditions. Even if the proposed protocol can be adapted for the traceability task, future work may consider image sample acquisition in less controlled conditions. In this case, deep learning-based methods can potentially be used to address the characterization problem (Goodfellow et al, 2016). Finally, the images used in this study were acquired using the same mobile camera. Future work may explore the model's generalization ability to different acquisition devices.

Author Declarations

Funding

This work was funded by the project *Generación de valor agregado en polen mediante la determinación de su origen botánico a través de métodos de aprendizaje supervisado* funded by Minciencias – Fondo nacional de financiamiento para la ciencia, la tecnología y la innovación Francisco José de Caldas.

Conflicts of interest/Competing interests

The authors declare that they have no conflicts of interest or competing interests related to this publication.

Consent to participate

Not applicable

Consent for publication

Not applicable

Availability of data and material/ Data availability

The data used in this study are available from the authors upon reasonable request.

Code availability

The code used in this study are available from the authors upon reasonable request.

Authors' contributions

JLC performed the experiments, acquired the data, and developed the code for analysis. CM contributed to the conceptualization, methodology design, and interpretation of results. FG also contributed to the conceptualization, methodology design, and interpretation of results. All authors participated in writing the manuscript, and all read and approved the final version.

References

Agashe SN (2006) Palynology and its application. Journal of Palaeosciences 55(1-3)):115–116

Akoglu H (2018) User's guide to correlation coefficients. Turkish journal of emergency medicine 18(3):91–93

Almeida-Muradian LBd, Sousa RJ, Barth OM, et al (2014) Preliminary data on brazilian monofloral honey from the northeast region using ft-ir atr spectroscopic, palynological, and color analysis. Química Nova 37:716–719

Arthur D, Vassilvitskii S (2006) How slow is the k-means method? Annual Symposium Computational Geometry 22

Astolfi G, Goncalves AB, Menezes GV, et al (2020) Pollen73s: An image dataset for pollen grains classification. Ecological Informatics 60:101165

Aung MM, Chang YS (2014) Traceability in a food supply chain: Safety and quality perspectives. Food control 39:172–184

Bertolini M, Bevilacqua M, Massini R (2006) Fmeca approach to product traceability in the food industry. Food control 17(2):137–145

Burger W, Burger J (2009) Principles of Digital Image Processing, fundamental tecniques. Springer

Celebi ME (2011) Improving the performance of k-means for color quantization. Image and Vision Computing 29(4):260–271

Devkota K, dos Santos CF, Raguse-Quadros M, et al (2022) Influence of seasonal weather variables and habitat type on numbers of colonies of the giant honey bee in nepal. Apidologie 53(1):3

Dhawale V, Tidke J (2013) Neural network based classification of pollen grains. In: 2013 International Conference on Advances in Computing, Communications and Informatics (ICACCI), IEEE, pp 79–84

Dimou M, Goras G, Thrasyvoulou A (2007) Pollen analysis as a means to determine the geographical origin of royal jelly. Grana 46(2):118–122

Donnelly KAM, Karlsen KM, Olsen P, et al (2008) Creating standardised data lists for traceability: a study of honey processing. International Journal of Metadata, Semantics and Ontologies 3(4):283–291

Dubois E (2010) The structure and properties of color spaces and the representation of color images, vol 11. Springer

Galhardo D, Garcia RC, Schneider CR, et al (2020) Microbiological quality of l. honey samples from western paraná, southern brazil. Journal of Apicultural Science 64(2):209–218

Gonçalves PJ, Estevinho LM, Pereira AP, et al (2018) Computational intelligence applied to discriminate bee pollen quality and botanical origin. Food chemistry 267:36–42

Goodfellow I, Bengio Y, Courville A (2016) Deep learning. MIT press

Grandini M, Bagli E, Visani G (2020) Metrics for multi-class classification: an overview. arXiv preprint arXiv:200805756

Gray J, Boucot A (1975) Color changes in pollen and spores: a review. Geological Society of America Bulletin 86(7):1019–1033

Haefeker W (2021) Pollen supplements and substitutes in the eu feed market: a product/market survey for bees and other animal species. EFSA Supporting Publications 18(2):6461E

Halbritter H, Ulrich S, Grimsson F, et al (2018) Methods in palynology. Illustrated Pollen Terminology pp 97–127

Jorgensen TH, Richardson DS, Andersson S (2006) Comparative analyses of population structure in two subspecies of nigella degenii: evidence for diversifying selection on pollen-color dimorphisms. Evolution 60(3):518–528

Katerinopoulou K, Kontogeorgos A, Salmas CE, et al (2020) Geographical origin authentication of agri-food products: A review. Foods 9(4):489

Kieliszek M, Piwowarek K, Kot AM, et al (2018) Pollen and bee bread as new health-oriented products: A review. Trends in Food Science & Technology 71:170–180

Klein AM, Vaissière BE, Cane JH, et al (2007) Importance of pollinators in changing landscapes for world crops. Proceedings of the royal society B: biological sciences 274(1608):303–313

Koo TK, Li MY (2016) A guideline of selecting and reporting intraclass correlation coefficients for reliability research. Journal of chiropractic medicine 15(2):155–163

Kostić AŽ, Milinčić DD, Barać MB, et al (2020) The application of pollen as a functional food and feed ingredient—the present and perspectives. Biomolecules 10(1):84

Kraft T, Valdés L, Zheng Y (2018) Supply chain visibility and social responsibility: Investigating consumers' behaviors and motives. Manufacturing & Service Operations Management 20(4):617–636

Kurek-Górecka A, Górecki M, Rzepecka-Stojko A, et al (2020) Bee products in dermatology and skin care. Molecules 25(3):556

Lau JA, Galloway LF (2004) Effects of low-efficiency pollinators on plant fitness and floral trait evolution in campanula americana (campanulaceae). Oecologia 141:577–583

Lunau K (1995) Notes on the colour of pollen. Plant Systematics and Evolution 198:235–252

Luykx DM, Van Ruth SM (2008) An overview of analytical methods for determining the geographical origin of food products. Food chemistry 107(2):897–911

Mahmood T, Choi J, Park KR (2023) Artificial intelligence-based classification of pollen grains using attention-guided pollen features aggregation network. Journal of King Saud University-Computer and Information Sciences 35(2):740–756

Nates-Parra G, Montoya PM, Chamorro FJ, et al (2013) Origen geográfico y botánico de mieles de apis mellifera (apidae) en cuatro departamentos de colombia. Acta biológica colombiana 18(3):427–438

Opara LU (2003) Traceability in agriculture and food supply chain: a review of basic concepts, technological implications, and future prospects. Journal of Food Agriculture and Environment 1:101–106

Otsu N (1979) A threshold selection method from gray-level histograms. 1 9

Paschos G (2001) Perceptually uniform color spaces for color texture analysis: an empirical evaluation. IEEE transactions on Image Processing 10(6):932–937

Porto RG, de Almeida RF, Cruz-Neto O, et al (2020) Pollination ecosystem services: A comprehensive review of economic values, research funding and policy actions. Food Security 12(6):1425–1442

Prdun S, Svečnjak L, Valentić M, et al (2021) Characterization of bee pollen: Physico-chemical properties, headspace composition and ftir spectral profiles. Foods 10(9):2103

Prince SJ (2023) Understanding Deep Learning. MIT press

Rodriguez-Damian M, Cernadas E, Formella A, et al (2006) Automatic detection and classification of grains of pollen based on shape and texture. IEEE Transactions on Systems, Man, and Cybernetics, Part C (Applications and Reviews) 36(4):531–542

Rünzel MA, Hassler EE, Rogers RE, et al (2021) Designing a smart honey supply chain for sustainable development. IEEE Consumer Electronics Magazine 10(4):69–78

Russell SJ (2010) Artificial intelligence a modern approach. Pearson Education, Inc.

Salazar-González CY, Rodríguez-Pulido FJ, Terrab A, et al (2018) Analysis of multifloral bee pollen pellets by advanced digital imaging applied to functional food ingredients. Plant Foods for Human Nutrition 73:328–335

Sanderson C, Wells H (2005) The flower fıdelıty of the honeybee. Uludağ Arıcılık Dergisi 5(1):32–41

Sculley D (2010) Web-scale k-means clustering. In: Proceedings of the 19th international conference on World wide web, pp 1177–1178

Silva B, Gonzaga LV, Maltez HF, et al (2021) Elemental profiling by icp-ms as a tool for geographical discrimination: The case of bracatinga honeydew honey. Journal of Food Composition and Analysis 96:103727

Thakur M, Nanda V (2020) Composition and functionality of bee pollen: A review. Trends in Food Science & Technology 98:82–106

Tsiknakis N, Savvidaki E, Manikis GC, et al (2022) Pollen grain classification based on ensemble transfer learning on the cretan pollen dataset. Plants 11(7):919

Uršulin-Trstenjak N, Puntarić D, Levanić D, et al (2017) Pollen, physicochemical, and mineral analysis of croatian acacia honey samples: applicability for identification of botanical and geographical origin. Journal of Food Quality 2017

Vadivel A, Sural S, Majumdar AK (2005) Human color perception in the hsv space and its application in histogram generation for image retrieval. In: Color imaging X: processing, hardcopy, and applications, SPIE, pp 598–609

Wang J, Zujovic J, Choi J, et al (2020) Influence of texture structure on the perception of color composition. J Percept Imaging 3(1):10401–1

Waser NM (1986) Flower constancy: definition, cause, and measurement. The American Naturalist 127(5):593–603

Waugh C, Clark G (2021) Factors affecting test reproducibility among laboratories. Revue Scientifique et Technique (International Office of Epizootics) 40(1):131–143

Wilcock C, Neiland R (2002) Pollination failure in plants: why it happens and when it matters. Trends in plant science 7(6):270–277

Yu Z, Jung D, Park S, et al (2022) Smart traceability for food safety. Critical Reviews in Food Science and Nutrition 62(4):905–916

Zhang YD, Yang ZJ, Lu HM, et al (2016) Facial emotion recognition based on biorthogonal wavelet entropy, fuzzy support vector machine, and stratified cross validation. IEEE Access 4:8375–8385
