# Supplementary material for: Classification of images of bee pollen according to their producers
Source: PLoS One. 2025 Oct 21;20(10):e0334615. doi: 10.1371/journal.pone.0334615 (PMC12539701; doi:10.1371/journal.pone.0334615)
Supplement: Attachment — Submitted filename: Response to Reviewers.pdf [file pone.0334615.s002.pdf]

PONE-D-24-51855

Classification of images of bee pollen according to their producers

Plos One

Dear Plos One Editor,

We thank the reviewers for all the thoughtful comments and suggestions that helped improve our work's presentation and clarity. In particular, we appreciate the very careful reading of our manuscript. We thank you for the comments and hope our modifications and responses fulfill the requirements. For ease of review, all our responses are marked in green, and the corresponding modifications in the manuscript appear in blue.

Reviewers' comments and responses:

Comment by reviewer

These comments were made directly on the pdf manuscript

Line 78: Time order!

Response: We thank the reviewer for pointing out the chronological ordering of the references on line 78. In the revised manuscript, we have adjusted the reference format to comply with the PLOS ONE guidelines.

Line 103: Not only does the acquisition process interfere with the color of the pollen, but also its production by the bees! How do you deal with that? Besides, the dry process (temperature) can interfere with the color of the pollen.

Response: We thank the referee for commenting on the possibility that 1) bees' pollen production and 2) pollen processing may interfere with the colorimetric pattern herein characterized. We agree with the referee about the need to clarify these points.

In our study, we focused on *Apis mellifera*, a species well known for its floral constancy during foraging. According to Sanderson and Wells (2005), Gonzalves et al. (2018a) and Heuel et al. (2024), once a foraging bee selects a particular floral source, it tends to continue collecting pollen from the same species over extended

periods. This behavior leads to reduced intra-colony variability in pollen origin and, consequently, a high degree of botanical uniformity in pollen samples. While we acknowledge that minor biochemical transformations can occur during pollen collection and packing by bees, the dominant influence on pollen color remains botanical origin, in fact, this is the main observation underlying the colorimetric work commonly devised in the palynology domain (Erdman (1969), Faegri (1989), Moore et al. (1991)). To address this point, we included the following statement in the manuscript in the *Materials and Methods* section (lines 142-146):

“Also, honeybee foraging behavior, particularly in Apis mellifera, is characterized by floral constancy, whereby worker bees consistently visit the same plant species once selected. This behavior contributes to reduce the variability in the botanical origin of collected pollen over time (Sanderson and Wells, 2005, Gonzalves et al., (2018a), Heuel et al., (2024)), thereby reducing color variation attributable to plant species.”

We agree that the drying process can potentially affect pollen color if not properly standardized. For this reason, all participating pollen producers followed a unified protocol (Vásquez et al. 2021), which includes precise drying parameters: stainless steel dryers, temperatures between 50 - 60 °C, and durations from 12 to 24 hours. For this study, all samples were dried for 24 hours at 55 °C, resulting in a final moisture content of 5%. This strict control minimizes variability caused by drying. We have now clarified this protocol in the *Materials and Methods* section (lines 147-158) as follows :

“For controlling variability in the pollen production, the producers involved in the study followed a standardized production protocol (Vásquez et al. (2021)). This protocol considered the drying method, specifying a temperature range of 50 to 60 degrees Celsius, using stainless steel machinery, and a drying duration of 12 to 24 hours to achieve a moisture content ranging from 5% to 8%. For this study, a drying time of 24 hours was applied to each sample, resulting in a final moisture content of 5%. Since the drying parameters were standardized, it is expected that the variability in color associated with this extrinsic characteristic will not have a significant impact. The pollen samples were maintained at a temperature of 55 C to facilitate grain manipulation. For each sample, 100 grams of dried pollen were manually spread across a non-reflective template, ensuring a uniform distribution that avoided both clumping and excessive empty space. This process was repeated before capturing each of the four images per sample, thereby reducing the influence of local dispersion artifacts and maximizing the representativeness of the sample’s color composition.”

Line 106: Do you mean the representation of each pollen type on the pollen spectrum?

Response: We thank the reviewer for identifying a potential ambiguity in this sentence. We agree that the expression "*each pollen species*" may have been misleading. To improve clarity, we have removed the wording in latest version of the manuscript.

Line figure 1 comment: Set them?

Response: We thank the reviewer for highlighting the importance of providing a more precise description of the image acquisition process. In response, we have added a detailed description of the parameters used to capture the macroscopic pollen images in the revised version of the manuscript. Specifically, we now state (lines 173-175):

"In this experiment, the images were captured using a frontal cellphone Xiaomi REDMI Note camera with 32 Mp, a proportion of 9:16, the option of high image quality, f = 2, s = 1/30 and 175 iso = 200."

Line 119: Only! Too few.

Response: We thank the reviewer for raising this important point related with the number of visits performed to acquire data. We recall that these visits aimed to characterize the temporal variability in pollen composition associated with regional flowering dynamics. The number of four sampling periods per year was determined based on a balance between the scientific goal and practical limitations, including logistical constraints and the economic feasibility of coordinating repeated collections with local producers.

To select these sampling windows, we conducted a preliminary survey of the flowering phenology in the region, complemented by input from producers and two local experts in beekeeping and native flora. This process ensured that the chosen periods captured meaningful ecological variation in pollen availability throughout the year.

In addition, the study was conducted in a tropical ecosystem of the Boyacá department (Colombia), where seasonal variation is less pronounced compared to temperate regions. As noted by Alexander von Humboldt Biological Resources Research Institute from Colombia (IIRBvH ,2020), many of the floral species frequented by bees in these environments bloom year-round or with overlapping phenophases, thereby reducing the need for high-frequency sampling to capture floristic turnover.

Also, it is worth noting that despite four visits being performed, a representative number of samples, between 20 and 30 per-beekeeper, was acquired at each visit, providing a more comprehensive view of the pollen composition to be characterized. These rationale are now clarified in the revised version of the manuscript, which includes the following paragraphs in the *Materials and Methods* section (lines 114-141):

“The samples were collected in 2021 from paramo and humid ecosystems during each flowering season to capture potential variations in bee preferences. This study involved beekeepers from various locations in the Márquez region, including the municipalities of Nuevo Colón, Turmequé, Viracachá, and Ciénega, and the Tundama region, encompassing the municipalities of Tibasosa, Tuta, Belén, Santa Rosa de Viterbo, Duitama, Paipa, and Tuta, all located within the Boyacá department of Colombia. These municipalities are located in regions known for high pollen production.

For the sample acquisition, a comprehensive survey was first conducted to determine the flowering periods of the plant species present in the ecosystems relevant to the project. This information was essential for subsequently identifying the origin of the pollen collected by the colonies in the participating apiaries. A one-year collection calendar was developed in collaboration with local beekeepers and two local experts in bee production systems with field experience comprising four distinct pollen sampling periods. To set this number of visits, the producers and two local experts in bee production systems with field experience considered the variations in the flowering cycle of the region under study. It is worth mentioning that in tropical areas as the region under study, in contrast to other regions, there are no marked seasons, and consequently, most plants visited by bees blossom the whole (Araújo Rodarte et al, 2022).. This factor contributes to reducing the floristic variability along the year, contributing to minimize the number of visits for collecting samples.

A total of twenty beekeepers were instructed to collect pollen samples biweekly during each period, resulting in a total of six collections over a three-month span. In each collection round, five hives were randomly selected. Provided the total number of hives allowed, no hive was to be sampled more than once per period. An average of 200 grams of pollen was collected per hive during each sampling event, and the samples were stored under refrigeration until retrieval. In each sampling period, a total of 30 samples per beekeeper were expected. This procedure was repeated during all four periods to account for the temporal variability; some challenges arose due to rainfall, which hindered pollen collection by the bees. As a result, in certain apiaries, only four of the six planned biweekly collections were completed, yielding 24 samples per period instead of the intended 30. The quantity of samples were recollected each time per producer is shown in

Figure 2, which presents the distribution of the number of pollen samples per producer across all visits."

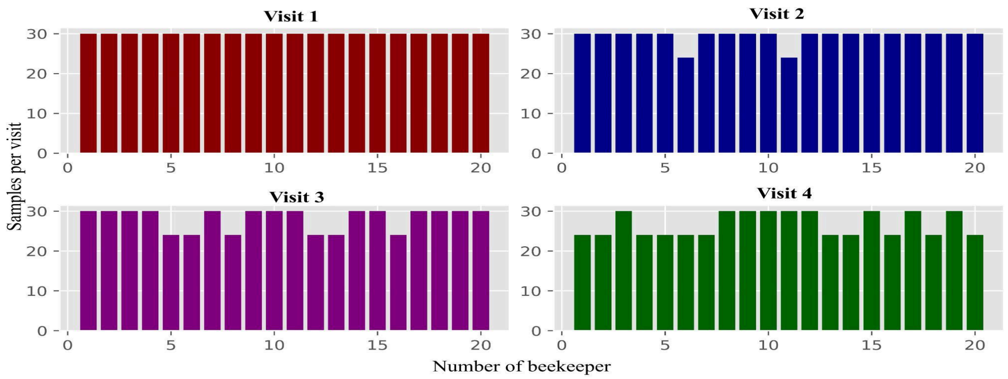

Figure 2: Frequency distribution of pollen samples per producer across the different sampling visits.

Line 143 Pollen grains:

Response: We thank the referee for point-out this typo. In this last version of the manuscript it was corrected to "pollen grains".

Line Fig 3 comment: There are many plants whose pollen grains, when dried, exhibit similar colors, even though they come from different plants. This factor needs to be considered.

Response: We thank the reviewer for pointing out that visually similar colors may emerge across pollen types after drying, even when grains originate from different plant species. This phenomenon is well known in classical palynology, where expert analysts rely on subjective visual assessments to characterize subtle pigment cues, often associated with sporopollenin and carotenoid content, for differentiating taxa based on color (Hornby et al., 2022).

Our approach addresses this characterization by capturing pollen color under standardized lighting conditions and transforming the raw RGB values into perceptually informative color spaces. This quantitative representation enables the classifier to identify chromatic variations that may not be discernible to the human eye.

Despite this standardization and color based characterization, we acknowledge that a small subset of pollen types may remain indistinguishable by color alone. To address this, future work could incorporate uncertainty estimation methods, such as conformal prediction (Angelopoulos & Bates, 2021), to assess the confidence of location predictions and guide downstream decisions accordingly. These

clarifications were added to the *Limitations* subsection of the *Discussion* section as follows (lines 502-509):

"This study demonstrates that quantitative color analysis can complement traditional morphological features by reducing the subjectivity associated with human color perception (Hornby et al., 2022), for instance, in the perception of similar colors, even when they come from different plants. Pollen grains were imaged under controlled illumination and converted into informative color spaces, enabling the classifier to capture subtle chromatic features associated with botanical origin. The variety of training samples also helps capture subtle color variations. However, in some cases, color alone may not suffice to distinguish between taxa. Future work may include confidence estimation frameworks, such as conformal prediction (Angelopoulos & Bates, 2021), to enhance interpretability and reliability of classification outcomes."

Line 170: Not always! Pollen types instead of pollen species.

Response: We thank the reviewer for pointing out the imprecise use of terminology. We agree that "pollen types" is more appropriate than "pollen species" in this context, as it better reflects the taxonomic resolution achievable in palynological studies. The text has been revised accordingly throughout the manuscript.

Line 280 and 288: Don't start your text this way, as the figure doesn't illustrate anything. Who illustrates are the authors, presenting their data in an image (the figure, the graph...). Rewrite your text.

Response: We thank the reviewer for this observation regarding the phrasing used in lines 280 and 288. We agree that the wording should clarify that it is the authors who present or explain the data using the figure, rather than the figure itself "illustrating" it. We have revised the sentences accordingly to reflect this distinction more accurately.

Line 321: Your data is correct, but I believe that the association between color and your pollen samples is only applicable in this specific case and for these particular samples. I believe that for samples from different periods, as there will be quantitative variation in the pollen spectrum, the association with color may be compromised. Perhaps if a study of the pollen spectrum had been conducted, color could be associated with the pollen types, and their quantity in the pollen samples could provide a more precise association. In any case, even if it does not work perfectly, your method could be improved to ensure greater confidence in the colorimetric association and also simplified so that it can be used by beekeepers or beekeepers' associations.

Response: We thank the reviewer for this annotation about the role of the pollen spectrum in strengthening the association between color and sample origin. As

noted, variations in the quantitative composition of pollen types across different periods could affect the reliability of color-based associations. However, to address this issue, our study considered both ecological characteristics of the region and temporal validation.

First, the region under study exhibits relatively low variability in plant composition throughout the year (IIRBvH ,2020). This is due to the tropical climate and the floral fidelity of Apis mellifera, which tends to forage consistently from the same plant species across seasons (Sanderson, 2005, Araújo et al., 2022). These factors reduce the likelihood of abrupt shifts in the pollen spectrum and support the temporal stability of the color associations.

Second, we acknowledge that environmental conditions, particularly oxidation, can affect pollen color during storage. To minimize this risk, all samples were processed under controlled drying and storage protocols, as outlined in (Vásquez , 2021)), which specify temperature, humidity, and light exposure parameters known to preserve color integrity.

Third, beyond these assumptions, we empirically evaluated the model's capacity to generalize across different time periods. As sampling was conducted in four distinct seasonal windows between October 2020 and November 2021, we used these temporal samples, for studying robustness of the proposed method when tested on different periods.

For this, we examined the model performance for different visits using the test partition. This approach allowed us to assess whether the color-based associations held across periods. Our empirical findings, presented in the last version of the manuscript in Table 1, suggest that the association between color and geographic origin remains consistent across different periods. This consistency was observed when the classification model was trained using data from all sampling periods, which implicitly incorporates the temporal variability of the pollen spectrum into the training process. Under these conditions, an explicit characterization of the pollen spectrum is not strictly required to achieve reliable classification performance.

However, we fully agree that including a detailed analysis of the pollen spectrum could provide additional insight and further strengthen the observed associations. For example, incorporating the distribution of pollen types as an intermediate representation might offer a more nuanced understanding of how specific botanical sources influence color patterns. Therefore, we propose the integration of this approach as part of our future work. This extension would not only improve the biological interpretability of the model but could also serve as a foundation for more robust and transparent tools, potentially facilitating practical applications such as traceability systems or decision-support tools for beekeepers and producers. In order to clarify these important points we performed the following modifications:

In the *Materials and Methods* section we added the following paragraph (lines 258-263):

“To examine the temporal generalizability of the classification model, the test partition was divided into four partitions corresponding to each of the seasonal sampling visits. In a temporal testing setup, the model was evaluated in the four visits, simulating prediction on an unseen time window. This approach allowed us to study the model’s ability to provide unbiased performance under temporal shifts in the pollen spectrum.”

In the Results section we added the following paragraph (lines 345-348):

“The results, presented in Table 1, show that the model maintained high accuracy, precision, and recall across all periods. These findings suggest that despite potential quantitative shifts in the pollen spectrum, the colorimetric patterns captured by the proposed classification strategy remain stable across time.”

In the *Limitation* subsection *Discussion* section we added the following paragraph (lines 256-465):

“The temporal variation in the botanical composition of pollen could potentially affect the association between color and sample identity. However, our results suggest that the colorimetric patterns captured by the model remain stable across different sampling periods, see Table 1. As previously discussed, this temporal robustness could be linked to the relatively low seasonal turnover in floral sources in the study region and the foraging fidelity of Apis mellifera. Nonetheless, the explicit characterization of the pollen spectrum, particularly its temporal dynamics, could further strengthen the model's interpretability and precision. Incorporating the information of the distribution of pollen types as an intermediate representation may provide additional insights into the underlying biological processes that drive color variation and can enhance the characterization of location. Therefore, we propose this intermedial characterization of pollen as a direction for future work.”

Table 1: Performance metrics of the model across temporal sampling visits

Visit	Balanced Accuracy	Recall (weighted)	Precision (weighted)	F1 Score (weighted)
1	0.88	0.88	0.88	0.87
2	0.84	0.84	0.85	0.84
3	0.78	0.78	0.80	0.78
4	0.75	0.74	0.75	0.73

Line 449: Too few studies on pollen color!

Response: We agree with the referee about the need of considering more studies on pollen color. Therefore, the following studies has been reviewed and considered in the last version of the manuscript: Bleha et al, 2021; Borlinghaus et al, 2024, Barbara 2015 (lines 74-89):

"Color information has been previously explored in pollen characterization. For example, Bleha et al. (2021) proposed a method to characterize bee pollen using visual classification, microscopy, and vibrational spectroscopy. They used CIE Lab* values to measure color, and Fourier Transform-Raman spectroscopy to identify pigment differences, especially in carotenoids and flavonoids, a multivariate analysis helped distinguish pollen types, showing the method's value for quality control and authentication. Borlinghaus (2024) examined bee pollen from several regions in Germany, identifying 14 major European pollen types. Pollen was classified using light microscopy, and color data were analyzed with Gaussian Mixture Models. The study found high color variation within species, often beyond human perception, highlighting the limitations of human color-based species identification. While these methods advance botanical description, they rely on microscopy or complex techniques and do not address geographical origin. Complementary studies show that regional differences affect the physicochemical and nutritional properties of bee pollen(Bárbara et al, 2015; Melo et al, 2023). Melo et al (2023) linked color, pH, protein, and fatty acid profiles to Brazilian regions. At the same time, Barbara et al (2015) found location-dependent variation in protein, fiber, lipid, and phenolic content in Melipona mandacaia pollen. These results support the use of chemical and color metrics to infer geographical origin."

Reviewer # 1

1/I am not convinced how the technique is consistent/reproducibility and can be applied in other areas. Apart from color differentiation, can any other parameters be considered as well?

Response: We thank the reviewer for raising the important issue of consistency, reproducibility, and general applicability of our method. We want to remark that for ensuring consistency and reproducibility, we employed a standardized and documented protocol for pollen collection, image acquisition, and storage conditions, minimizing variability due to environmental or handling factors. Additionally, the image analysis pipeline was fully automated, and the model was trained and validated using cross-validation and temporal partitioning strategies. These measures allowed us to assess the model's robustness.

For clarifying these points, the last version of the manuscript provides a highly detailed description of all steps required to reproduce the proposed approach. In

particular: the second section outlines the procedures required to reproduce the proposed model, sub-section 2.1 describes the experimental design used for data collection, along with contextual information regarding the origin of the data, in sub-section 2.2, the tools developed for the study are introduced, as well as the standardized protocol applied to both the acquisition of representative data and the photographic capture process, sub-section 2.3 provides an overview of the preprocessing methods employed, sub-section 2.4 presents the theoretical foundations underlying the use of color as a means of data representation. The subsequent sections propose various classification models, which can be adapted in future researchers aiming to reproduce this process.

In addition, in order to guarantee computational reproducibility of the results we also included all the computer code and the complete dataset required for reproducing the reported results. This information reads as follows in the last version of the manuscript in the *Materials and Methods* section (lines 303):

“The used code and the image database needed to reproduce results can be accessed in https://github.com/JuanDavid1703/PolenProject.git.”

Related with the application to other areas, we acknowledge that generalization to other geographic regions or botanical contexts may require additional adaptations. In regions with greater floral diversity or different beekeeping practices, the relationship between color and pollen origin may vary. In addition, we included a novel subsection discussion the main points related to reproducibility in *Discussion* section as follows (lines 434 - 454):

“4.3 Reproducibility

The proposed method shows promising results in classifying bee pollen samples by producer using color information. However, we recognize the importance of evaluating its reproducibility and its potential applicability beyond the specific conditions of this study. The potential for applying this approach to other locations, particularly in tropical regions, is supported by several factors. First, Apis mellifera exhibits floral fidelity (Lau and Galloway, 2004; Jorgensen et al, 2006), consistently foraging on the same plant species once it has been selected. This behavior may contribute to low intra-annual variability in pollen botanical composition, a feature that enhances consistency in pollen appearance across time and among nearby producers. Second, tropical ecosystems are characterized by continuous or overlapping flowering cycles for most plant species (Instituto de Investigación de Recursos Biologicos Alexander von Humboldt, 2020), which reduces seasonal variability in pollen sources. These ecological factors support the stability of color-based pollen characteristics, allowing for adequate sampling at regular intervals throughout the year. In addition to these environmental factors, the study implemented a standardized pollen production protocol that producers in other regions can adopt. By maintaining consistency in pollen collection, drying, and imaging acquisition and processing, variability introduced by processing is minimized, contributing to reproducibility. Additionally, by training on a broad set of

reference samples, including those from different flowering periods, the model captures both intra- and inter-taxon color variation. Finally, to evaluate model robustness, we employed both cross-validation and temporal partitioning strategies, testing the model on data from different flowering periods. Considering all these factors in other locations should contribute to the reproducibility of the pattern described here."

To enhance generalizability and reduce reliance on color alone, future work may incorporate complementary features, including, texture descriptors (e.g., local binary patterns or Gabor filters) to capture surface characteristics, morphological features derived from microscopic or macroscopic grain analysis, and spectral data obtained through hyperspectral or near-infrared imaging to capture chemical and structural variations not visible in RGB images. For accounting for this relevant point we include in the *Discussion* section the following text (lines 423-433):

"Finally, we acknowledge that color represents only one of the potentially discriminative features suitable to describe pollen origin. To enhance the generalizability of classification models, future research could integrate complementary features beyond color. For instance, computational analysis of surface texture may help distinguish visually similar samples, while grain morphology, extracted from macro- or microscopic images, can reveal taxonomically relevant traits. In this case, mobile phone-based microscopy (Switz et al, 2014) may also offer again a low-cost, accessible tool for acquiring such data in field conditions. Additionally, incorporating spectral information from hyperspectral or near-infrared imaging could capture chemical and structural attributes beyond the RGB spectrum, potentially improving classification performance (Sendin et al, 2018). However, these advanced techniques may be less feasible for widespread deployment across the production chain, and their integration should consider cost and accessibility constraints."

In addition, beyond bee pollen characterization, the approach has potential for adaptation to other domains. For example, provenance verification in honey, propolis, or wax by analyzing visual or spectral traits associated with origin, quality control in agricultural commodities (e.g., coffee, spices, or seeds), where visual or physical characteristics are critical indicators of geographic or botanical origin, traceability tools for food authentication, particularly in natural products where certification of origin impacts market value. We thank the referee for calling our attention to these highly relevant potential areas of application, which are now considered in the Discussion section (381-388).

"Beyond the specific case of bee pollen, the proposed methodology may be adapted to other domains where product traceability and origin verification are critical. For example, similar image-based approaches could be applied to honey, propolis, or wax, using visual or spectral patterns to identify producers or regions (Shafiee et al, 2014). In agriculture, this technique could support quality control in commodities such as coffee, seeds, or spices, where physical and visual

characteristics often reflect geographic origin and cultivation practices (Zhu et al, 2021). Applications may also extend to food authentication, particularly in the natural products industry, where origin labeling plays an important role in consumer trust and market value (Wu et al, 2021)."

2/Can the author show at least two different locations to show elements of reproducibility?

Response: We thank the reviewer for the suggestion to include results from more than one location to assess reproducibility. Due to logistical and budgetary constraints, this study was limited to a single geographic region within the department of Boyacá (Colombia). We agree that extending the method to additional locations would strengthen generalizability and reproducibility. However, our approach aligns with well-established practices in the field, where initial validation is often performed in a single location to control variability and demonstrate feasibility. Studies such as Malinowski et al. (2021), Zheleznyak et al., 2021, and Estevinho et al. (2017) similarly developed and tested characterization methods using samples from a single site. These works are frequently cited and serve as precedent for the current study's design.

We have added a paragraph in the Discussion section to acknowledge this context and clarify how future work will address multi-site validation.

"The study was conducted in a single geographic region within the department of Boyacá, Colombia (see Figure 7). Logistical and budgetary constraints primarily drove the decision to focus on this area. However, the proposed methodology was designed to be reproducible and scalable, with standardized protocols at each stage of data collection and analysis. It is worth recalling that while the classification model in this study was trained and tested using data from producers in a single region, the conditions under which the method was developed, floral fidelity, and low temporal pollen variability are not exclusive to the area under study and are commonly found across other tropical beekeeping zones (Amaya, 2009; Amakpe et al, 2024). However, we recognize the importance of evaluating the generalizability of this approach beyond the current context. Therefore, future work will involve collaboration with producer associations in other tropical regions to assess the model's performance in new environments, validate its robustness, and explore the integration of additional features such as texture or spectral information to improve classification in more diverse ecological settings.

It is worth noting that many studies in the fields of palynology, food traceability, and spectroscopic classification have focused on single geographic regions when developing and validating new methodologies. This approach is prevalent in early-stage or feasibility studies, where logistical and ecological consistency is essential to isolate key variables and establish robust protocols. For example, Swiatly-Blaszkiewicz et al (2021) characterized bee pollen from a single region in west-central Poland using ED-XRF and ATR-FTIR spectroscopy, achieving consistent elemental and organic profiles across samples. Similarly, Fourier

Transform Infrared Spectroscopy and Raman spectroscopy have been used effectively to classify pollen types from a single location based on vibrational signatures (Kendel and Zimmermann, 2020). Anjos et al (2017) also applied Fourier Transform Infrared - Attenuated Total Reflectance spectroscopy to 126 bee pollen samples from a single Portuguese region to assess compositional traits and verify botanical origin. These studies demonstrate that scientifically rigorous and reproducible classification systems can be developed within localized contexts."

Finally, while our results are specific to a set of producers in one region, the methodology was designed with controlled, standardized, and reproducible components, making it adaptable to other tropical regions with similar ecological and production characteristics. Specifically, our approach relies on (1) the floral fidelity of Apis mellifera, which results in low inter-annual variability in pollen composition; (2) the continuous flowering cycles typical of tropical ecosystems, which allow for consistent pollen collection throughout the year; and (3) a standardized protocol for pollen collection, drying, imaging, and processing. These conditions are not unique to the Boyacá region and can be reasonably replicated by producers across other tropical areas, as discussed in section Reproducibility in the last version of the manuscript.

Reviewer #2

Remember to consider some aspects of pollen biology and bee behavior that may influence the coloration of the collected pollen. Additionally, there are external factors, such as the drying method, which can also interfere with the coloration.

Response: We highly appreciate your valuable observations about the importance of considering pollen biology, bee behaviour and pollen preparation as possible sources of changes of color. We agree with the referee about the importance of considering these elements.

In our study, we focused on *Apis mellifera*, a species well known for its floral constancy during foraging. According to Heuel et al (2024); Gonzalves et al (2018a); Sanderson and Wells (2005), once a foraging bee selects a particular floral source, it tends to continue collecting pollen from the same species over extended periods. This behavior leads to reduced intra-colony variability in pollen origin and, consequently, a high degree of botanical uniformity in pollen samples. While we acknowledge that minor biochemical transformations can occur during pollen collection and packing by bees, the dominant influence on pollen color remains botanical origin, in fact, this is the main observation underlying the colorimetric work commonly devised in the palynology domain (Erdman 1969, Faegri 1989, Moore et al. 1991). To address this point, we included the following statement in the manuscript in the *Materials and Methods* section:

“It is worthy recall that honeybee foraging behavior, particularly in Apis mellifera, is characterized by floral constancy, whereby worker bees consistently visit the same plant species once selected. This behavior, supported by Heuel et al (2024); Gonzalves et al (2018a); Sanderson and Wells (2005), results in minimal variability in the botanical origin of collected pollen over time, thus reducing color variation attributable to plant species,particularly those that provide the most nutrients for the hive (Waser, 1986).”

In addition, we acknowledge that the pollen nature is an essential determinant of the color information herein characterized. Therefore, we also included a complete description of the rationale underlying the pollen sample collection, emphasizing the primary role of color stability of pollen in tropical regions. These changes read as follows in the Materials and Methods section (lines 115-130):

“This study involved beekeepers from various locations in the Márquez region, including the municipalities of Nuevo Colón, Turmequé, Viracachá, and Ciénega, and the Tundama region, encompassing the municipalities of Tibasosa, Tuta, Belén, Santa Rosa de Viterbo, Duitama, Paipa, and Tuta, all located within the Boyacá department of Colombia. These municipalities are located in regions known for high pollen production.

For the sample acquisition, a comprehensive survey was first conducted to determine the flowering periods of the plant species present in the ecosystems relevant to the project. This information was essential for subsequently identifying the origin of the pollen collected by the colonies in the participating apiaries.A one-year collection calendar was developed in collaboration with local beekeepers and two local experts in bee production systems with field experience comprising four distinct pollen sampling periods. To set this number of samples, the producers and two local experts in bee production systems with field experience considered the variations in the flowering cycle of the region under study. It is worth mentioning that in tropical areas as the region under study, in contrast to other regions, there are no marked seasons, and consequently, most plants visited by bees blossom the whole (Araújo et al. 2022). This factor contributes to reducing the floristic variability along the year, helping to minimize the number of visits for collecting samples.

We agree that the drying process can potentially affect pollen color if not properly standardized. For this reason, all participating pollen producers followed a unified protocol based on Vásquez et al. (2021), which includes precise drying parameters: stainless steel dryers, temperatures between 50–60 °C, and durations from 12 to 24 hours. For this study, all samples were dried for 24 hours at 55 °C, resulting in a final moisture content of 5%. This strict control minimizes variability

caused by drying. We have now clarified this protocol in the *Materials and Methods* section as follows (lines 147-158):

"The producers involved in the study followed a standardized production protocol for the pollen production (Vásquez et al. (2021)). This protocol considered the drying methods, specifying a temperature range of 50 to 60 degrees Celsius, using stainless steel machinery, and a drying duration of 12 to 24 hours to achieve a moisture content ranging from 5% to 8%. For this study, a drying time of 24 hours was applied to each sample, resulting in a final moisture content of 5%. Since the drying parameters were standardized, it is expected that the variability in color associated with this extrinsic characteristic will not have a significant impact. The pollen samples were maintained at a temperature of 55°C to facilitate grain manipulation. For each sample, 100 grams of dried pollen were gently spread across a non-reflective template, ensuring a uniform distribution that avoided both clumping and excessive empty space. This process was repeated before capturing each of the four images per sample, thereby reducing the influence of local dispersion artifacts and maximizing the representativeness of the sample's color composition."

Reviewer #3

1. The method is based on the hypothesis that each producer / region has a distinct color trait. The authors should explain in more detail the cause of these color patterns. As stated in page 7, "a relationship exists between the color of the pollen and the species from which it originates". Is pollen color constant for a certain species?

Response: We thank the reviewer for this insightful comment about the need to motivate the relationship between color and species/location. The idea that color can serve as a proxy for botanical or geographical location originates from well-established practices in palynology, where the initial step in pollen analysis frequently involves visual separation of grains by color (Erdman, 1969; Moore et al., 1991; Faegri, 1989). In our case, this rationale is also supported by the floral constancy behavior of Apis mellifera, which leads foragers to repeatedly visit the same floral species during a foraging bout (Heuel et al., 2024; Sanderson & Wells, 2005; Gonçalves et al., 2018). This behavioral trait promotes botanical uniformity within pollen loads, thereby reinforcing the representativeness of color as a compositional signal.

However, we acknowledge that pollen color is not strictly constant within a species. Color variability can result from several factors, including pigment concentration, plant maturity, environmental conditions, and slight physiological variations. Despite this, our results show that the colorimetric patterns captured under

standardized imaging conditions exhibit high predictive power, even when intra-species variability is present.

We also acknowledge that the biological mechanisms behind these observed color differences, particularly how regional floral assemblages shape the chromatic composition of collected pollen, are complex and likely extend beyond the scope of this paper. Nonetheless, our work contributes empirical evidence supporting the hypothesis that pollen color, as captured in digital images, encodes useful information for origin discrimination, even at the producer level within a relatively homogeneous region. For addressing this point more explicitly in the manuscript, we have added a new sub-section the *Discussion* section to overcome these points (lines 395-439):

"Color as a discriminative feature in pollen

This study demonstrates that macroscopical color information provides a reliable basis for classifying bee pollen according to its producer. The proposed model builds upon the well-established role of color in palynological studies (Almeida-Muradian et al, 2014; Halbritter et al, 2018) and extends previous work by demonstrating its applicability to producer-level classification. The observed non-random distribution of pollen colors (see Figure 8) aligns with ecological factors such as plant community composition and pollinator fidelity behavior (Lau and Galloway, 2004; Jorgensen et al, 2006), reinforcing the idea that color carries meaningful geographical and botanical information, as observed in the associations observed in Figure 8). In this case, interestingly, some misclassifications occurred between producers closely located (see Figure 7 for an illustration), suggesting a strong geographical signal in the color patterns acquired at the macroscopical level.

In contrast to earlier efforts that rely on microscopic imaging or physicochemical analyses (Gonc¸alves et al, 2018a; Astolfi et al, 2020; Tsiknakis et al, 2022; Mahmood et al, 2023), our approach focuses on more accessible macroscopic image data, seeking to reduce technical barriers for real-world applications, such as food traceability. By employing a robust and compact color-based representation space, we can effectively capture discriminative features even at a macro-scale acquisition. This relationship is enhanced by considering a controlled acquisition protocol, post-preprocessing to mitigate acquisition artifacts, and a probability-based clustering representation, which proved to be essential for maximizing the quality of the extracted information. The results highlight the potential of color information from macroscopical images as a practical and scalable tool for producer-level pollen classification, opening new possibilities for automated systems in the context of apicultural quality control and traceability based on color.

It should be emphasized that pollen color can vary within a species due to physiological and environmental factors. Our results show that digital images acquired under standardized conditions contain sufficient chromatic information to support origin discrimination at the producer level, see Figure 8 and Table 1. However, the underlying biological mechanisms explaining these patterns are complex and require further investigation. Despite this limitation, our findings provide the first quantitative support for using color as a proxy in pollen origin classification. We recognize that applying this method to other regions or ecosystems may require additional validation. In particular, differences in botanical composition and environmental conditions could affect the relationship between color and origin. However, the presence of certain key factors, such as floral fidelity of bees, vegetation stability, well-characterized flowering periods, standardized image pollen collection protocols, and the effectiveness of color-based machine learning algorithms, may facilitate the identification of analogous patterns in new contexts.

Finally, we acknowledge that color represents only one of the potentially discriminative features suitable to describe pollen origin. To enhance the generalizability of classification models, future research could integrate complementary features beyond color. For instance, computational analysis of surface texture may help distinguish visually similar samples, while grain morphology, extracted from macro- or microscopic images, can reveal taxonomically relevant traits. In this case, mobile phone-based microscopy (Switz et al, 2014) may also offer again a low-cost, accessible tool for acquiring such data in field conditions. Additionally, incorporating spectral information from hyperspectral or near-infrared imaging could capture chemical and structural attributes beyond the RGB spectrum, potentially improving classification performance (Sendin et al, 2018). However, these advanced techniques may be less feasible for widespread deployment across the production chain, and their integration should consider cost and accessibility constraints

2. Following on question 1, does the approach assume that the combination of plant species is unique for every producer or region? Is this always the case?

Response: We appreciate this follow-up question, which raises an important point about the assumptions underlying our approach. The proposed method does not assume that each producer or region is associated with a unique combination of plant species. This would indeed be an unrealistic assumption, especially in cases where apiaries share similar ecological zones, floristic composition, or are part of the same agroecosystem.

Instead, our hypothesis is that subtle yet systematic differences in foraging behavior, microenvironmental conditions, and hive-specific dynamics result in variations in the relative abundance and composition of pollen types collected at different sites. These variations can produce distinct colorimetric profiles, even if

the overall floral pool is broadly shared. This is particularly plausible given the floral fidelity of Apis mellifera and the fine-scale differences in resource availability and hive-level foraging preferences (Gonçalves et al., 2018; Heuel et al., 2024).

While such differentiation may be less pronounced in highly homogeneous regions, our results demonstrate that, under standardized image acquisition conditions, these subtle chromatic variations are sufficient to enable successful origin discrimination at the producer level. In more heterogeneous ecological contexts, such as comparisons across climatic zones (e.g., temperate vs. tropical regions), the discriminatory power of colorimetric features is likely enhanced.

To clarify this in the manuscript, we have added the following statement to the *Discussion* section:

"...the approach does not rely on the assumption that each producer has access to a completely distinct floral assemblage. Rather, it captures subtle differences in the composition and proportion of pollen types collected by different hives, which are influenced by microenvironmental variation, local management practices, and hive-specific foraging patterns. These differences are reflected in consistent colorimetric patterns that can be detected and modeled for origin classification."

3. Section 2, Sample Image Acquisition: The authors write that "the grains were evenly spaced". How is this spreading performed? How could it affect the acquired images and the classification result?

Response: We thank the reviewer for this question about the need to clarify this procedure. The phrase "evenly spaced" refers to the uniform distribution of the pollen grains across the surface of the photographic template, rather than precise geometric spacing. This distribution was achieved manually, by spreading a standardized quantity of 100 grams of dried pollen over a non-reflective surface. The aim was to avoid both clumping of grains, which could result in overlapping colors or shadowing artifacts, and large empty areas, which would reduce adequate sample coverage in the image.

This spreading procedure was developed through several preliminary trials to identify the amount and dispersion pattern that produced maximal chromatic diversity while maintaining visual clarity. The uniform distribution ensures that the dominant color patterns of the sample are adequately represented and that the classifier receives balanced visual input.

To further reduce the impact of potential local inconsistencies in distribution, four images were taken per sample, each after remixing and redistributing the pollen. This strategy aimed to minimize the risk of introducing bias due to uneven

coverage or clumped areas in a single image. We have clarified this procedure in the revised version of the manuscript as follows (lines 154-158):

"For each sample, 100 grams of dried pollen were manually spread across a non-reflective template, ensuring a uniform distribution that avoided both clumping and excessive empty space. This process was repeated before capturing each of the four images per sample, thereby reducing the influence of local dispersion artifacts and maximizing the representativeness of the sample's color composition."

4. Section 2, Sample Image Acquisition: The authors should give more details on the camera used in the experiments. At the end of the Discussion section, they mention the generalization to other devices as future research, but they don't give details on the camera or the minimum technical requirements needed to replicate their experiments.

Response: We thank the referee for this comment about the technical requirements needed to replicate the experiment. For overcoming this point, the following features of the camera are added to the *Sample image acquisition* subsection in the *Materials and Methods* section (lines 173-175):

"In this experiment, the images were captured using a frontal cellphone Xiaomi REDMI Note camera with 32 Mp, a proportion of 9:16, the option of high image quality, f = 2, s = 1/30 and 175 iso = 200."

5. Section 2.5., Color feature extraction: The number of centroids for each producer is determined by the number of samples detected in a palynological analysis. Is this number maintained constant for each producer?

Response: We thank the reviewer for this clarification request about the number of centroids in the clustering analysis. While the initial palynological analysis identified a variable number of floral groups per producer, ranging from 16 to 35, this variability was not directly used to define the number of centroids per producer. Instead, the palynological findings served as an empirical reference to determine a plausible range for the number of representative colors (centroids) needed to capture the chromatic diversity in each sample.

For ensuring methodological consistency and comparability across all samples, a fixed number of centroids was used for all producers during the feature extraction process. Specifically, after conducting a series of validation experiments that treated the number of centroids as a hyperparameter, we selected a value that maximized classification performance while preserving generalizability.

This clarification has now been included in the revised manuscript in the *Materials and Method* section as follows as follows (lines 242-249):

“Although palynological analysis revealed that the number of floral types per producer ranged from 16 to 35, this variability was not used to define producer-specific color clustering. Instead, a fixed number of centroids was applied to all samples to ensure comparability and standardization. This value was selected after cross-validating performance across the tested range.”

6. Section 2.6: The authors indicate that “various multiclass classification strategies were explored” that included support vector machines and perceptrons. But the method used to obtain the final results presented in Section 3 is not specified. For the sake of reproducibility, the authors should:

a. Indicate what was the actual method that gave the results presented in the paper.

Response: We thank the reviewer for emphasizing the importance of specifying the method used in the final results. The model that performed best in the study was a Support Vector Machine (SVM) trained on color features extracted in the Luv color space, using 32 representative color centroids per sample. The SVM employed a 6th-degree polynomial kernel, a regularization constant (C) of 0.91, and an independent coefficient (coef0) of 1.3. We have now clarified this in the Results section of the manuscript with the following description:

“The best results were obtained using a SVM classifier trained with *32* representative color centroids extracted from the *Luv* color space projected to the *uv* dimension. The SVM was configured with a *6th-degree* polynomial kernel, a regularization parameter $C=0.91$, and an independent coefficient $c_0=1.3$.”

b. The input vector has a different size for each region, depending on the number of species detected. How is this difference handled in the machine learning algorithm?

Response: We thank the reviewer for this observation. We want to clarify that the objective of this work was not to identify botanical species, but rather to predict the geographical origin (i.e., the producer/location) of pollen samples using image-based color features.

Although the initial palynological analysis provided an estimate of the floral types per producer, this information was not used to determine the input vector size. Instead, to ensure consistency across all samples and locations, we fixed the number of representative color centroids at 32 for all pollen samples, regardless of the number of botanical types detected.

Therefore, each image was thus processed to extract a feature vector of fixed size, composed of the 32 representative color centroids projected into various color spaces (e.g., RGB, HSV, Luv). This ensured that all input vectors fed into the

classifiers had the same dimensionality, avoiding inconsistencies during model training. We have revised the manuscript to explicitly include this point in the *Materials and Methods* section (lines 246-249):

"Since the number of floral species may vary, the analysis of this parameter revealed that using 32 color centroids provided the best representation across all samples, this standardization ensured that each input vector had the same dimensionality, independent of regional species variations."

c. Give details on the implementation of the method (parameters that need to be tuned, etc.). Only a final best result is provided in the paper. What was the variability in the results with different machine learning techniques?

Response: We appreciate the request for more detailed information regarding the implementation of the classification models and the variability observed during experimentation. To identify the most effective configuration, we performed an extensive evaluation combining different classification algorithms, color feature extraction parameters, and hyperparameter settings.

In particular, three supervised machine learning algorithms were tested: Support Vector Machines (SVM), Multilayer Perceptrons (MLP), and Random Forests (RF). For each algorithm, we explored approximately 20 combinations of hyperparameters (e.g., kernel type and degree for SVM; architecture, learning rate, and activation functions for MLP; number of trees and maximum depth for RF). In parallel, we varied the number of color centroids used to represent each image—from 16 to 35 centroids—based on the range of botanical groups detected in prior palynological analyses. These centroids were extracted from each image using k-means clustering in different color spaces.

In total, this resulted in the training and evaluation of approximately 1,200 models, allowing us to systematically examine the interaction between classifier type, input dimensionality, and hyperparameter choices.

Regarding performance, the Support Vector Machine consistently produced the best results across evaluation metrics, achieving F1-scores between 0.80 and 0.85. The Random Forest classifier showed moderate performance, with typical F1-scores in the range of 0.70 to 0.78. In contrast, the Multilayer Perceptron exhibited substantial variability and generally underperformed, with F1-scores dropping as low as 0.56 in some configurations. Across all models, increasing the number of centroids tended to improve performance slightly, although the influence of the algorithm type was more significant than the number of centroids. These changes reads as follows in the *Results* section (lines 327-330):

“Regarding performance, the SVM consistently produced the best results across evaluation metrics, achieving F1-scores between 0.80 and 0.85. The Random Forest classifier showed moderate performance, with typical F1-scores in the

range of 0.70 to 0.78. In contrast, the Multilayer Perceptron exhibited substantial variability and generally underperformed, with F1-scores dropping as low as 0.56 in some configurations. Across all models, increasing the number of centroids tended to improve performance slightly, although the influence of the algorithm type was more significant than the number of centroids."

7. Section 2.7, Experimental evaluation. The authors should describe the distribution of producers among the final set of samples used in the experiments.

Response: We thank the reviewer for this important observation about the need to complement the description of samples per producer. To address this point, we have included a detailed summary of the distribution of samples per producer in the revised version of the manuscript. Specifically, we added a new figure (Figure 2) in the Materials and Methods section that presents a frequency distribution diagram showing the number of samples collected and used for validation per producer.

This figure provides a clear overview of the data balance among the 20 participating producers, helping to contextualize the experimental design and the evaluation of model performance. The revised manuscript now reads as follows in the *Materials and methods* section (lines 139-141):

"The quantity of samples were collected each time per producer is shown in Figure 2, which presents the distribution of the number of pollen samples per producer across all visits."

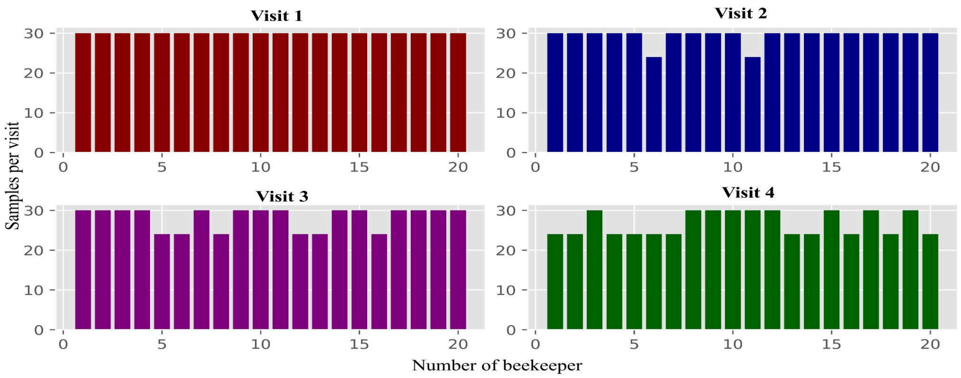

Figure 2: Frequency distribution of pollen samples per producer across the different sampling visits.

8. From a translational point of view: If a new producer enters the market, the system needs to be retrained to include the new producer. The authors should comment on this in the Discussion section.

Response: We thank the reviewer for this valuable observation. We have now added the following paragraph to the Discussion section to address this translational consideration explicitly:

"It is important to note that the classifier developed in this study is trained on a fixed set of producers. Therefore, when a new producer should be included for the characterization of its location, the proposed model should be retrained to incorporate samples from that producer into the classification framework (Sculley et al (2015)). While this requirement does imply an additional step for extending the system, it does not undermine the validity or utility of the approach presented here. We recall that our primary contribution lies in demonstrating that a relatively simple, accessible, and reproducible image-based method can effectively classify corbicular pollen samples by origin under standardized conditions. The results serve as proof of concept that color-based features extracted from macroscopic images hold strong discriminatory power for pollen origin. Looking forward, this approach may be adapted to classify samples at broader geographical scales, such as ecological zones or regional denominations of origin, rather than individual producers, which could enhance its scalability and reduce the need for frequent retraining."

Bibliography:

- Angelopoulos, A. N., & Bates, S., (2021). A gentle introduction to conformal prediction and distribution-free uncertainty quantification. CoRR, abs/2107.07511. https://arxiv.org/abs/2107.07511.
- Anjos, O., Santos, A. J. A., Dias, T., & Estevinho, L. M. (2017). *Application of FTIR-ATR spectroscopy on the bee pollen characterization. Journal of Apicultural Research*, 56(3), 210–218. https://doi.org/10.1080/00218839.2017.1289657
- Araújo Rodarte, A.T., Rodríguez, C., Muniz, M., Verzosa, A., Campos, L., de oliveira, P., Ávila, T., Alves, E. (2022). Flowering Phenology in a *Restinga* Community: 7 Years of Study. In: Medeiros, M.F.T., de Sá Haiad, B. (eds) Aspects of Brazilian Floristic Diversity . Springer, Cham. https://doi.org/10.1007/978-3-031-07453-0_1.
- Bárbara, M. S., Machado, C. S., Sodré, G. D. S., Dias, L. G., Estevinho, L. M., & De Carvalho, C. A. L. (2015). Microbiological Assessment, Nutritional Characterization and Phenolic Compounds of Bee Pollen from *Mellipona mandacaia* Smith, 1983. *Molecules*, vol *20* (7), pages 12525-12544. https://doi.org/10.3390/molecules200712525.
- Bleha, R., Shevtsova, T. V., Živčáková, M., Korbářová, A., Ježková, M., Saloň, I., Brindza, J., & Synytsya, A. (2021). Spectroscopic Discrimination of Bee Pollen by Composition, Color, and Botanical Origin. *Foods*, vol *10* (8), page 1682. https://doi.org/10.3390/foods10081682.
- Borlinghaus, P., Tausch, F., & Odemer, R. (2024). Natural color dispersion of corbicular pollen limits color-based classification. *ISPRS Open Journal of Photogrammetry and Remote Sensing,* vol *12*, Article 100063. https://doi.org/10.1016/j.ophoto.2024.100063.
- Instituto de Investigación de Recursos Biológicos Alexander von Humboldt. (2020). Colección Herbario Federico Medem Bogotá - FMB. 114009 registros. http://i2d.humboldt.org.co/ceiba/resource.do?r=herbario_instituto_humboldt.
- Jardine, P. E. and Fraser, W. T. and Lomax, B. H. and Gosling, W. D. (2015). The impact of oxidation on spore and pollen chemistry, Journal of Micropaleontology, vol 34, pages 139-149. https://doi.org/10.1144/jmpaleo2014-022.
- Goncalves, P. J., Estevinho, L. M., Pereira, A.P. (2018). Computational intelligence applied to discriminate bee pollen quality and botanical origin. Food chemistry, vol 267: pages 36–42.
- Hornby, S., Benn, J., Vinkenoog, R., Goldberg, S., & Pound, M. J. (2022). Methods in melissopalynology: colour determination of pollen pellets for colour vision deficient individuals. *Palynology*, vol *46* (4), pages 1–7. https://doi.org/10.1080/01916122.2022.2062476.

- Heuel, K. C., Haßlberger, T. A., Ayasse, M. (2024). Floral trait preferences of three common wild bee species. Insects, vol 15.
- Sanderson C, Wells H, The flower fidelity of the honeybee. Uludag Bee Journal, vol 5, page 32–41, 2005, Bursa Uludağ University.
- Hornby, S., Benn, J., Vinkenoog, R., Goldberg, S., Pound, M. J.(2022). Methods in melissopalynology: colour determination of pollen pellets for colour vision deficient individuals. *Palynology*, vol 46 (4): pages 1–7. doi: https://doi.org/10.1080/01916122.2022.2062476.
- Kenđel, A., & Zimmermann, B. (2020). Chemical analysis of pollen by FT-Raman and FTIR spectroscopies. *Frontiers in Plant Science, 11*, Article 352. https://doi.org/10.3389/fpls.2020.00352 .
- Vásquez, R., Cepeda, M., Bejarano, D., Gutiérrez, A., Burgos, W., Rodríguez, O. (2021) Producción de polen, protocolo de buenas prácticas apícolas. Journal *buenas prácticas apícolas*, AGROSAVIA. https://editorial.agrosavia.co/index.php/publicaciones/catalog/view/241/222/1483-1.
- Moore, P. D., Webb, J. A., & Collinson, M. E. (1991). *Pollen analysis* (2nd ed.). Blackwell.
- Erdman, G. (1969). Handbook of palynology. An introduction of the study of pollen grains and spores. *Denmark: Copenhagen*.
- Faegri, K., Kaland, P. E., & Krzywinski, K. (1989). Textbook of pollen analysis.
- Wu, W., Zhang, A., van Klinken, R. D., Schrobback, P., & Muller, J. M. (2021). Consumer trust in food and the food system: a critical review. Foods, vol 10, page 2490.
- Sendin, K., Williams, P. J., & Manley, M. (2018). Near infrared hyperspectral imaging in quality and safety evaluation of cereals. Critical reviews in food science and nutrition, vol 58(4), pages 575-590.
- Świątły-Błaszkiewicz, A., Pietkiewicz, D., Matysiak, J., Czech-Szczapa, B., Cichocka, K., & Kupcewicz, B. (2021). Rapid and accurate approach for honeybee pollen analysis using ED-XRF and FTIR spectroscopy. *Molecules, 26*(19), 6024. https://doi.org/10.3390/molecules26196024.
- Waser, N. M. (1986). Flower constancy: Definition, cause, and measurement. *The American Naturalist, 127*, 593–603. https://doi.org/10.1086/284503
