# Supplementary material for: Classification of images of bee pollen according to their producers
Source: PLoS One. 2025 Oct 21;20(10):e0334615. doi: 10.1371/journal.pone.0334615 (PMC12539701; doi:10.1371/journal.pone.0334615)
Supplement: Attachment — Submitted filename: Response to Reviewers_26092025.pdf [file pone.0334615.s003.pdf]

PONE-D-24-51855R1

Classification of images of bee pollen according to their producers

Plos One

Dear Plos One Editor,

We thank the reviewers for all the thoughtful comments and suggestions that helped improve our work's presentation and clarity. In particular, we appreciate the very careful reading of our manuscript. We thank you for the comments and hope our modifications and responses fulfill the requirements. For ease of review, all our responses are marked in green, and the corresponding modifications in the manuscript appear in blue.

Reviewers' comments and responses:

Reviewer #1: General Comments:

1.There are always limitations of color-based identification on pollen.

We thank the referee for this highly valuable comment. We acknowledge that while color may present some limitations when establishing a digital fingerprint of the botanical origin of a pollen sample, the findings of this study demonstrate that, using color information acquired on standardized conditions, it is possible to predict its geographical origin based on color. To address this problem, two local experts in bee production worked with the producers to develop a “floral calendar” that documents flowering dynamics throughout the year. Moreover, because this is a tropical region, most plant species flower continuously, which reduces seasonal variability. These factors support the validity of our findings and provide a baseline for future research.

As discussed in the manuscript, for the present study, the available economic and logistical resources enabled analysis within a one-year range. A natural extension of this methodology would involve broadening the temporal scope and incorporating additional variables, such as nutritional composition or laboratory-derived features, while maintaining a favorable cost–benefit ratio to ensure feasibility for beekeepers with limited resources. Such work would allow us to verify whether significant color variation arises in relation to botanical origin beyond the conditions studied here.

2.Therefore, identification based solely on pollen color is limited, as color can vary within a single species and may also overlap across different species. As such, it does not provide a precise or reliable basis for pollen classification.

We appreciate this important observation. Pollen color can indeed vary within a species and overlap across different species, which limits its precision for species-level identification. However, it is important to clarify that the main objective of our study was not to identify the botanical species present in the samples, but rather to classify the producer. In this context, our results indicate that the proposed methodology achieves a reasonable level of accuracy for this task.

We fully recognize the inherent limitations of relying solely on color information, and this was precisely part of the research question: given that this is a cost-effective and accessible approach, how well can it perform in predicting the sample origin at the producer level? The results obtained so far suggest that it provides an appropriate predictive performance for this specific classification problem. Future work will naturally extend this framework to incorporate complementary features for species-level identification.

.

3. More advanced methods, such as pollen DNA metabarcoding, have been widely demonstrated by many researchers to be effective in tracing both the geographical and botanical origins of pollen.

We appreciate your suggestion regarding the use of DNA metabarcoding, as it is indeed a powerful and widely recognized method for tracing both the botanical and geographical origin of pollen. Nevertheless, one of the aims of our work is to propose a cost–time efficient alternative. In Colombia, and particularly within the beekeeping sector and pollen production chain, there is currently limited economic capacity to implement DNA-based methodologies or other resource-intensive analytical processes.

Recent advances in machine learning and computer vision have shown that color, when captured and analyzed with appropriate models, can serve as a reliable digital fingerprint. This work contributes to this literature. Although imperceptible to the human eye, subtle chromatic patterns can be effectively detected through accessible tools such as smartphones or cameras, enabling accurate predictions at a fraction of the cost. For example, whereas DNA metabarcoding services typically range between USD 100–250 per sample (University of North Texas, 2024–2025), the approach presented here operates at less than USD 1 per sample after model deployment, highlighting its practical advantages for producers with limited resources.

It is also important to note that DNA metabarcoding is not yet commonly available in Colombia and in many tropical regions. Beyond cost, there are technical barriers: many primers used in global metabarcoding protocols are not optimized for the high biodiversity of Colombian flora, and numerous local species remain underrepresented

in existing reference libraries. These constraints limit the immediate applicability of DNA barcoding in our context, as confirmed by local experts in bee science.

Nonetheless, we fully recognize the value of DNA-based methods as a complementary tool. In the revised manuscript, we explicitly acknowledge DNA metabarcoding as a promising direction for future work, while emphasizing that our current proposal offers a cost-effective, accessible, and scalable alternative that can already support traceability in developing regions..

The following comment will be added to the document:

Another area to explore is the characterization of pollen samples through DNA barcoding analysis. Studies have demonstrated that this technique is a powerful tool for determining both the geographical and botanical origin of honey samples, relying on the analysis of pollen residues (Ruppert, et al, 2019; Ullah and Huyop, 2024) . However, one limitation of this method should be noted: despite its effectiveness, the associated costs are not easily affordable (University of North Texas, 2024–2025).

4. Generally, Bee pollen is widely recognized for its health benefits, and its nutritional and bioactive properties varying by botanical origin.

We thank the referee for this important observation. In our study, we acknowledged that pollen is a key resource for sustaining bee colonies and that it also provides essential nutritional properties beneficial to humans (Oliveira et al., 2009). A promising research direction would be to investigate whether the botanical origin and nutritional composition of a sample can be inferred from its color characteristics, given that compounds such as isoflavonoids and carotenoids are simultaneously linked to pollen pigmentation and nutritional value (Velásquez et al., 2025; Rzepecka-Stojko et al., 2015). In this regard, the potential of color analysis to serve as a proxy for botanical origin and associated nutritional traits represents an attractive avenue for future work (Gámbaro et al., 2025).

Reviewer #2 General comments:

1. I recommend accepting the manuscript after language editing by a native speaker to improve some passages that may be poorly placed, but this consideration is also from someone who is not a native English speaker.

Thank you for your suggestions. The document has been shared with a native scientific language editor for proper review and style correction. The revisions were highlighted in blue, consistent with the approach previously applied to the paragraphs appended to the article. Thanks for your observations.

References:

1. Simão C. et al. (2025). Digital image processing + ML as an effective approach for bee pollen classification.
2. University of North Texas. (2025). *Genomics Center: Pricing*. UNT Research. https://research.unt.edu/resources/research-core-facilities/genomics-center/pricing.html.
3. Velásquez, P., Muñoz-Carvajal, E., Grimau, L., Bustos, D., Montenegro, G., & Giordano, A. (2023). Floral Pollen Bioactive Properties and Their Synergy in Honeybee Pollen. Chemistry & biodiversity, 20(4), e202201138. https://doi.org/10.1002/cbdv.202201138
4. Rzepecka-Stojko, A., Stojko, J., Kurek-Górecka, A., Górecki, M., Kabała-Dzik, A., Kubina, R., Moździerz, A., & Buszman, E. (2015). Polyphenols from Bee Pollen: Structure, Absorption, Metabolism and Biological Activity. Molecules, 20(12), 21732-21749. https://doi.org/10.3390/molecules201219800
5. Oliveira, K. C. L. S., Moriya, M., Azedo, R. A. B., Almeida-Muradian, L. B., Teixeira, E. W., Alves, M. L. T. M. F., & Moreti, A. C. C. (2009). Relationship between botanical origin and antioxidants vitamins of bee-collected pollen. *Química Nova, 32*(5), 1099-1102. https://doi.org/10.1590/S0100-40422009000500003
6. Gámbaro, A., Miraballes, M., Urruzola, N., Kniazev, M., Dauber, C., Romero, M., Fernández-Fernández, A. M., Medrano, A., Santos, E., & Vieitez, I. (2025). Physicochemical Composition and Bioactive Properties of Uruguayan Bee Pollen from Different Botanical Sources. Foods, 14(10), 1689. https://doi.org/10.3390/foods14101689
7. Ruppert, K. M., Kline, R. J., & Rahman, M. S. (2019). Past, present, and future perspectives of environmental DNA (eDNA) metabarcoding: A systematic review in methods, monitoring, and applications of global eDNA. *Global Ecology and Conservation, 17,* e00547. https://doi.org/10.1016/j.gecco.2019.e00547
8. Ullah, S., & Huyop, F. (2024). Using pollen DNA metabarcoding to trace geographical origin of honey; all identified taxa were indigenous to the region. *Meta Gene, 38,* 101012. https://doi.org/10.1016/j.mgene.2024.101012
